# Label-free streamlined photoacoustic image guidance facilitates NIR-II photoablation in models of melanoma lung metastases

**DOI:** 10.1172/JCI196095

**Published:** 2026-05-01

**Authors:** Wei Xing, Yujia Zhou, Katja Haedicke, Chenyixin Wang, Karla Ximena Vazquez-Prada, Hong Wu, Zhijun Lin, Chrysafis Andreou, Qize Zhang, Ke Shang, Ruoyang Hu, Moritz Kircher, Xingdong Ye, Jan Grimm, Jiang Yang

**Affiliations:** 1 State Key Laboratory of Oncology in South China, Guangdong Provincial Clinical Research Center for Cancer, and; 2 Department of Anesthesiology, Sun Yat-sen University Cancer Center, Guangzhou, China.; 3 Department of Cardiology, The First Affiliated Hospital, Jiangxi Medical College, Nanchang University, Nanchang, China.; 4 Department of Radiology, Memorial Sloan Kettering Cancer Center, New York, New York, USA.; 5 Department of Electrical and Computer Engineering, University of Cyprus, Nicosia, Cyprus.; 6 Department of Oncology, Xinyang Central Hospital, Xinyang, China.; 7 Institute of Dermatology, Guangzhou Dermatology Hospital, Guangzhou Medical University, Guangzhou, China.

**Keywords:** Dermatology, Oncology, Pulmonology, Cancer, Diagnostic imaging, Melanoma

## Abstract

Integrative multiscale imaging bridges the gap between macroscopic organ structures and microscopic cellular processes, enabling holistic visualization of anatomy and function across scales. Photoacoustic imaging (PAI) leverages melanin’s potent contrast for label-free melanoma detection, yet its potential in lung imaging, challenged by air-tissue acoustic impedance mismatch, remains unexplored for melanoma lung metastases (MLMs). We used hierarchical multiscale PAI, transitioning from whole-body macroscale to localized mesoscale and single-cell-resolution microscale. PAI also guided photoablation interventions in the first and second near-infrared windows, requiring only 10.4 pg intracellular melanin/cell. Bioinformatic analysis of human MLM tissues revealed perturbed signaling pathways compared with normal skin and lung tissues, accounting for dysfunctional melanogenesis to enable label-free PAI with high sensitivity and specificity. Malignant MLM lesions in living mice, resected mouse lungs, and human lungs were delineated with margins closely conforming to histology. The high sensitivity allowed visualization of low-cellularity microsatellite foci down to a few tens of cell clusters, with sufficient penetration in the lungs of mice and Bama minipigs. The multiscale imaging methodology streamlines a theranostic workflow and specifically identifies MLM burden in a progressive, label-free manner, which may aid real-time tumor ablation in the future.

## Abstract 2

Label-free multiscale photoacoustic imaging techniques to guide photoablation in the second near-infrared window to treat melanoma lung metastases without requiring the injection of extrinsic agents

## Graphical abstract

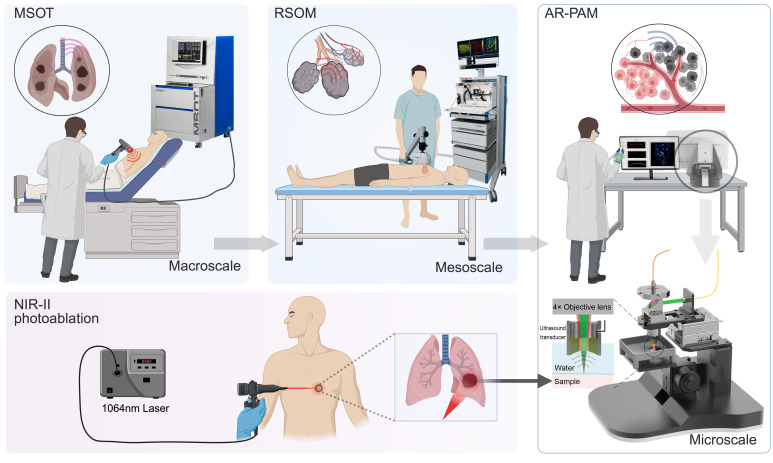

## Introduction

With high aggressiveness and rising yearly incidence, melanoma ranked as the fifth most common malignancy in the United States in 2023, causing more deaths than any other cutaneous cancer. Although emerging advances in immunotherapy and targeted therapy have slightly decreased the mortality rate of melanoma, recent statistics from the Surveillance, Epidemiology, and End Results database indicate that the 5-year relative survival rate for distant metastasis remains at 35%, with a poor prognosis — much lower than that of localized melanoma, which has a 5-year relative survival rate of greater than 99% (1). Melanin from melanoma can interfere with diagnostic histology by obscuring tumor cell details, masking staining patterns, and confounding morphology (2), mandating Fontana-Masson staining and high-resolution microscopic imaging. Among all visceral metastases, the lungs are the most common distant site, with a higher frequency (18%–36%) than the liver (14%–20%), brain (12%–20%), and bone (11%–17%), which often complicates treatment with a poorer prognosis and underscores the need for timely detection (3). However, early-stage pulmonary micrometastases are small and undetectable in the lung parenchyma until they progress into larger symptomatic lesions. Standard diagnostic imaging modalities, including chest X-ray, CT, and PET, are routinely used to visualize the extent of lung metastases, but all these modalities are associated with radiation exposure as a concern (4). Importantly, the air-tissue interface in air-filled lungs causes impedance mismatch and artifacts, remaining an enduring challenge for ultrasonography (US) but to a lesser extent for photoacoustic imaging (PAI). Fluorescence imaging has been evaluated for image-guided resection and biopsy confirmation in patients with ground-glass pulmonary nodules, demonstrating clinical success with OTL38 and VGT-309 imaging agents (5–10). Under intraoperative circumstances, extrinsic agents such as indocyanine green and FITC are often required but are challenged by limited imaging depth and high false-positive rates, even with targeting functionalities (11–13).

PAI is a hybrid modality that harnesses high-resolution optical contrast with ultrasonic penetration to greater depths. It optically excites and acoustically detects endogenous and exogenous optical contrast through nonradiative relaxation and thermoelastic expansion. The unmet need for clinical diagnostics with high sensitivity and specificity has been extensively explored recently using PAI (14–19). Due to the increased transparency of sound in biological tissues, PAI partially overcomes the limitations of scattering, achieving scalable spatial resolution at centimeter depths in both preoperative and intraoperative settings (13, 20–22). Although optical-resolution photoacoustic microscopy affords cellular resolution, acoustic-resolution photoacoustic microscopy (AR-PAM) with a spherically focused transducer allows deeper tissue penetration for in vivo imaging (23). Although chest-wall thickness depends on multiple factors, such as age, anatomic location, and BMI, cohort studies reveal an average thickness of a few centimeters along the anterior axillary and midclavicular lines (24, 25). An earlier study used indocyanine green contrast-enhanced PAI to locate indeterminate pulmonary nodules in tissues at depths of tens of millimeters (26).

Taking advantage of melanin’s broad light absorption that extends into the near-infrared (NIR) region, versatile PAI techniques have been implemented in human cutaneous melanoma to evaluate tumor depth, resection boundaries, angiogenesis, histopathology, sentinel lymph node metastases, and circulating melanoma cells in a noninvasive, nonionizing, near real-time, and label-free manner (27–32). Meanwhile, handheld portable systems at video frame rates have also been explored in multiple clinical studies, including breast cancer, sentinel lymph nodes, metabolism, and malformed vasculature (27, 33–35). PAI can also substitute high-cost pulsed lasers with more accessible laser diodes, overdriving light-emitting diodes, and xenon flash lamps for bedside diagnosis (36).

**Figure 1:**
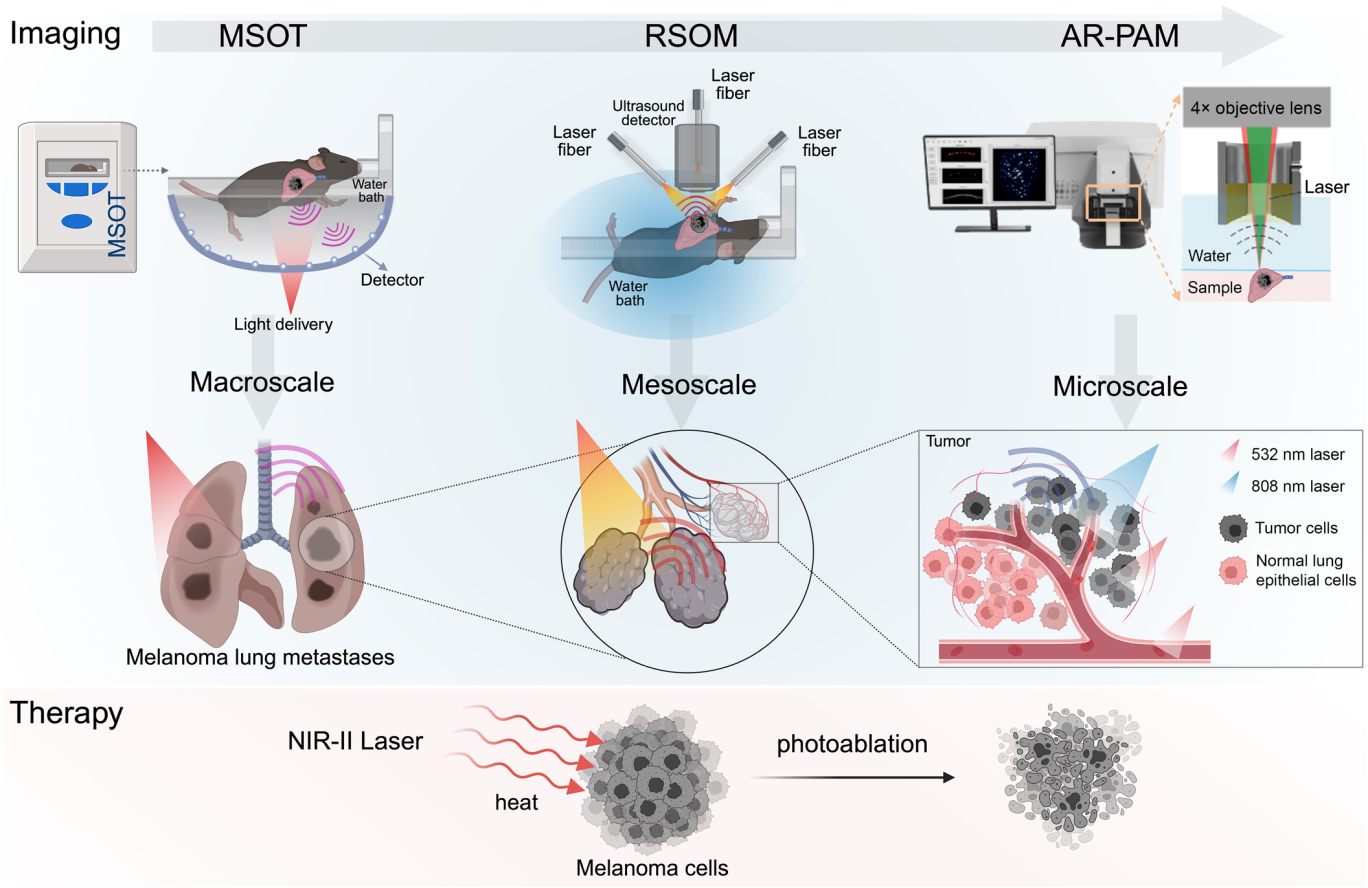
Concept of the pipelined, label-free, multiscale PAI-guided NIR-II photoablation therapy for MLMs. As a preliminary screening procedure, MSOT with a large FOV can penetrate the chest wall and thoracic diaphragm, providing panoramic views through 3D reconstruction of cross-sectional body-slice scans. Next, mesoscale RSOM is used to visualize localized tumor vasculature, hemorrhage, and clotting in lung nodules through divided high- and low-frequency US bands. Microscopically spread small lesions with only tens of melanoma cells can be sensitively and specifically detected in histopathological resolution by AR-PAM equipped with dual visible and NIR lasers. After a confirmatory diagnosis, noninvasive photoablation can be practiced to reduce MLM burdens at relatively low power in the NIR-II region.

To our knowledge, previous clinical and preclinical studies with endogenous melanin-enhanced PAI primarily focused on hyperpigmented benign nevi, cutaneous primary melanoma, and melanoma LN metastasis but lacked investigative attempts on melanoma lung metastases (MLMs) with label-free theranostics (30, 37–39). Here, we report a pilot theranostic study on PAI in MLM using a streamlined, hierarchical, photoacoustic workflow that progresses from whole-body macroscale tomography to localized mesoscopy to high-resolution microscopic configurations (Figure 1).

Although in situ MLM lesions in living mice are distinctly visualized by multispectral optoacoustic tomography (MSOT) at centimeter depths, which yields a high correlation with contours from histology and reporter gene-directed bioluminescence imaging (BLI), microscopic melanoma foci at 100 μm^2^ levels are distinguishable in high resolution from surrounding noncancerous lung tissues using AR-PAM techniques. A low limit of detection of 38.3 live B16-F10 cells/μL PBS was achieved in phantoms at a signal-to-noise ratio of 3. Notably, human MLM lesions from 3 patients were precisely identified by AR-PAM in histopathological resolution in surgically resected lung tissues. We leveraged the naturally melanotic features of MLM in lungs and directed noninvasive, noncontact photothermal ablation in the murine MLM allograft model through the thoracic diaphragm. Specifically, ablation at 1,064 nm in the NIR-II region outperformed NIR-I at 808 nm, achieving better therapeutic outcomes at identical laser power densities or comparable efficacy at a much lower power density. This stratified, multiscale PAI workflow, with complementary configurations, allows clinicians to start with a broad overview of MLMs and progressively focus on malignant areas of concern, optimizing imaging time and resources to improve the accuracy of therapeutic planning during perioperative periods for translation.

## Results

### Dysregulated melanogenesis signaling in MLMs.

**Figure 2:**
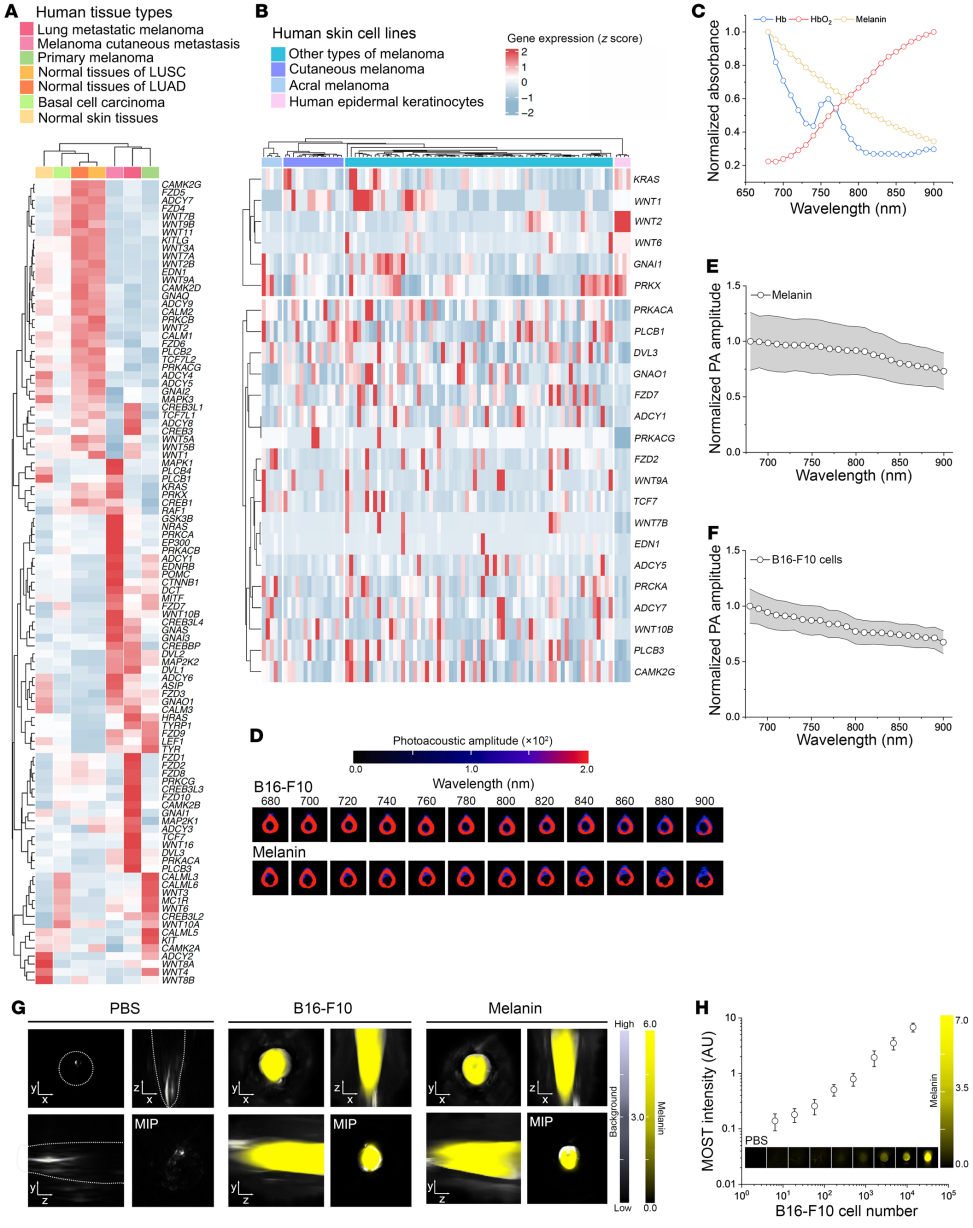
Dysregulated melanogenesis signaling in cutaneous melanoma and MLMs enables melanin-directed sensitive and specific photoacoustic detection. (**A**) Gene expressions involved in the melanogenesis pathway of the Kyoto Encyclopedia of Genes and Genomes database for human cutaneous melanoma, MLMs, peritumoral nontumor skin, peritumoral lung tissues of squamous cell carcinoma, and lung adenocarcinoma. (**B**) Melanoma-related gene expression profiling of human skin cell lines with differential expression (*P* < 0.05). (**C**) Comparison of absorption spectra between melanin and other intrinsic photoabsorbers of oxygenated (HbO_2_) and deoxygenated Hb in biological tissues, allowing for molecular imaging by spectral unmixing. (**D**) Wavelength-dependent photoacoustic phantom images of 1.4 × 10^4^ B16-F10 cells/μL. Photoacoustic spectra of (**E**) melanin and (**F**) B16-F10 melanoma cells. (**G**) Unmixed MSOT phantom images of PBS, B16-F10 cells, and melanin in 3D: *x*-*y*, *x*-*z*, and *y*-*z*. Maximum intensity projection (MIP) visualizations are displayed in the lower right corner. Unmixing is conducted using the factory-preset melanin-specific optoacoustic spectrum. (**H**) Linear correlation between the B16-F10 cell density and MSOT signal intensity (*R*^2^ = 0.988). Data are displayed in a log-log scale with the PBS control value subtracted.

Through gene analysis of human tissues of primary melanoma, MLMs, melanoma cutaneous metastasis, basal cell carcinoma (BCC), peritumoral normal lung tissues of lung squamous cell carcinoma (LUSC) and lung adenocarcinoma (LUAD), and normal skin tissues, we identified distinct differential expression patterns of melanogenesis in MLM tissues from other comparators (Figure 2A), thereby enabling label-free detection of melanoma tumor mass over a low-melanin lung tissue background. Key enzymes and regulators involved in melanin biosynthesis were found to be upregulated, such as microphthalmia-associated transcription factor, dopachrome tautomerase, tyrosinase, and tyrosinase-related protein 1 encoded by *MITF*, *DCT*, *TYR*, and *TYRP1* genes, respectively, along with effectors in the Wnt and MAPK pathways known to modulate melanocyte differentiation and proliferation (40). Interestingly, signaling by frizzled (*FZD*) genes was hyperactivated in MLM tissues compared with primary and cutaneous metastatic melanoma. Similarly, we profiled the melanogenesis panel in human melanoma cell lines. Various categories of melanoma cells exhibit differential expression patterns from normal human epidermal keratinocytes (Figure 2B). Though not fully understood, the melanogenesis machinery plays a complex role in regulating cutaneous melanoma progression, invasion, and metastasis.

### Multi-wavelength noninvasive MSOT detection of MLMs at the macroscale.

The perturbed melanin production in melanoma, with a unique, broad, continuous absorption across the visible-NIR wavelengths, facilitates its identification from interfering biological photoabsorbers, such as oxyhemoglobin (HbO_2_) and hemoglobin (Hb), via multispectral unmixing via MSOT (Figure 2C). In the meantime, phantom images of B16-F10 cells obtained at single wavelengths closely resemble those of melanin, exhibiting photoacoustic spectra that align with their corresponding absorption profiles (Figure 2, D–F). Likewise, 3D, reconstructed phantom images of B16-F10 cells and synthetic melanin presented comparable MSOT signals, which were undetectable in the PBS control (Figure 2G). Gradient concentrations of live B16-F10 melanoma cells exhibited a strong linear correlation (*R*^2^ = 0.988) with MSOT intensities in vitro (Figure 2H), yielding a low detection limit of approximately 6.4 cells/μL, close to our previous result (41).

**Figure 3:**
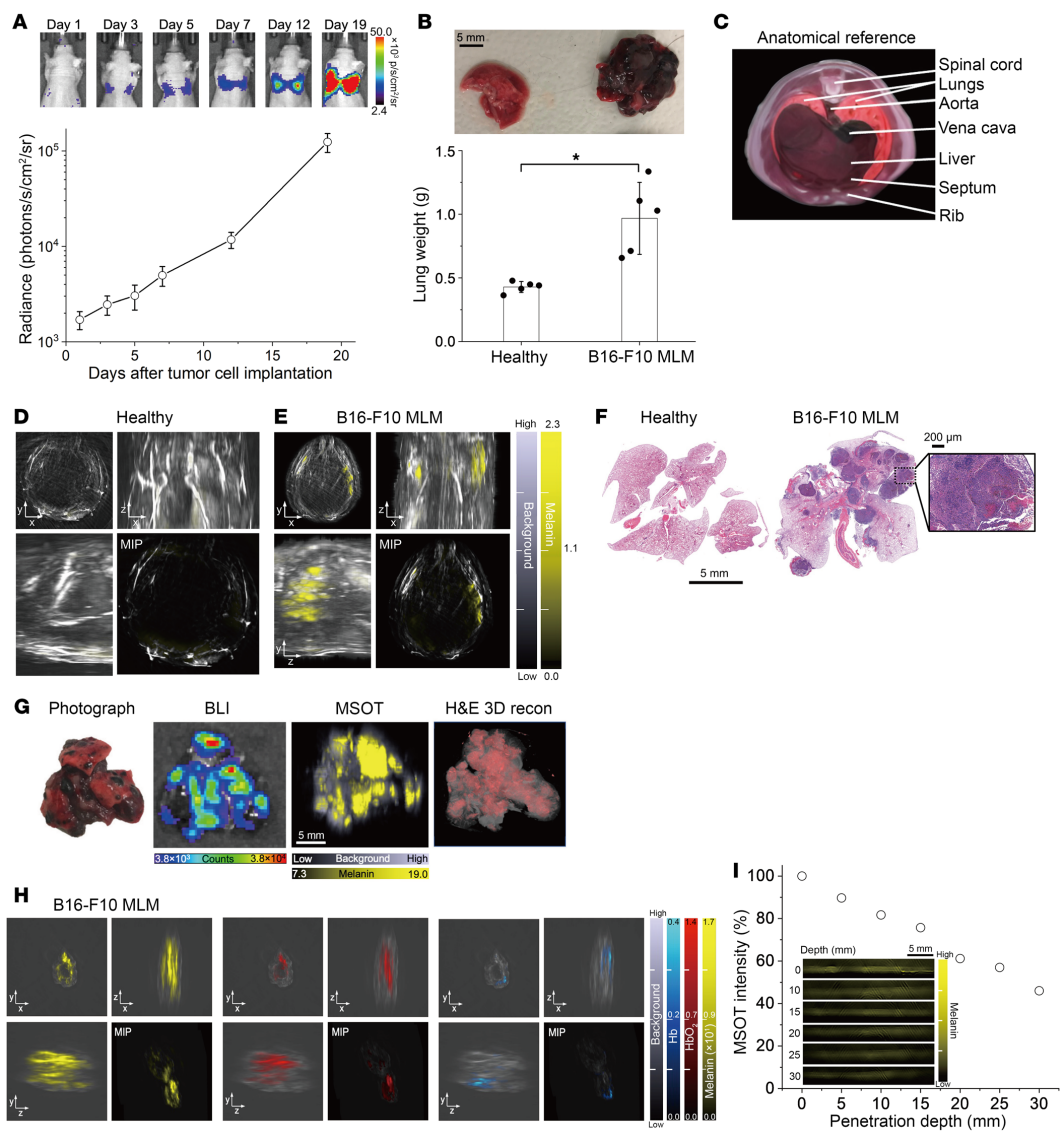
Macroscale tomographic detection of MLM lesions in living mice by MSOT. (**A**) Establishment of MLM mouse models by intravenous injection of murine B16-F10-Luc cells. Metastatic melanoma growth patterns in the lungs were tracked dynamically using BLI. (**B**) Organ weight of healthy and MLM lung tissues harvested from mice 19 days after implantation (*n* = 5). *P < 0.05 by 2-tailed Student’s *t* test. (**C**) Cross-sectional atlas from ex vivo mouse cryosection as an anatomic reference. Noninvasive MSOT imaging of (**D**) healthy and (**E**) B16-F10 MLM mouse models. Maximum intensity projection (MIP) images are shown in the lower right corners. Associated unmixed mapping images for Hb and HbO_2_ are provided in Supplemental Figure 1. (**F**) Representative histology of healthy and MLM lung tissues by H&E staining. An enlarged region shows typical tumor histopathology and intratumoral melanin deposition. (**G**) Aligned photograph, BLI, 3D reconstructed MSOT, and 3D H&E images of MLM lung tissues. (**H**) Ex vivo MSOT images of MLM lung tissues spectrally unmixed for melanin, Hb, and HbO_2_. (**I**) MSOT intensities of 2 × 10^4^ B16-F10 cells/μL in 2% agarose gel phantoms at different imaging depths.

To verify whether sensitive melanoma detection is feasible in vivo through the chest wall, we established MLM mouse models by intravenously injecting B16-F10 cells through the tail vein. After preferentially colonizing the lungs to mimic the hematogenous metastatic progression, melanoma colonies (modeling metastases) developed over time in the lung, as dynamically tracked by BLI (Figure 3A). Subsequently, the collected MLM tissues were heavier with dense nodules on visual inspection (Figure 3B). At the macroscale, we panoramically scanned the upper body using PAI, which houses the lungs in the thoracic cavity and includes the liver below the diaphragm in the abdominal cavity (Figure 3C). Melanoma-specific MSOT signals were negligible in healthy mice compared with B16-F10 MLM mice, in which metastatic cancer lesions were distinctly observable in a noninvasive, 3D tomographic fashion (Figure 3, D and E), confirmed by histopathology (Figure 3F). The imaging findings highlight the sufficient tissue penetration depth of MSOT through the chest wall and intact thoracic diaphragm for detecting MLM. More importantly, the reconstructed 3D MSOT projection aligned well with BLI and 3D H&E images in the context of MLM malignancies, underscoring its high reliability (Figure 3G). Leveraging the multispectral imaging features of MSOT, we could also map the distribution of HbO_2_ and Hb ex vivo and in vivo (Figure 3H and Supplemental Figure 1; supplemental material available online with this article; https://doi.org/10.1172/JCI196095DS1). Notably, the oxygenation state of melanoma in the lungs is heterogeneous with both hypoxic and normoxic areas. The imaging depth was estimated to be approximately 3 cm in vitro (Figure 3I).

### Label-free raster-scan optoacoustic mesoscopy of MLM vasculature characteristics at the mesoscale.

**Figure 4:**
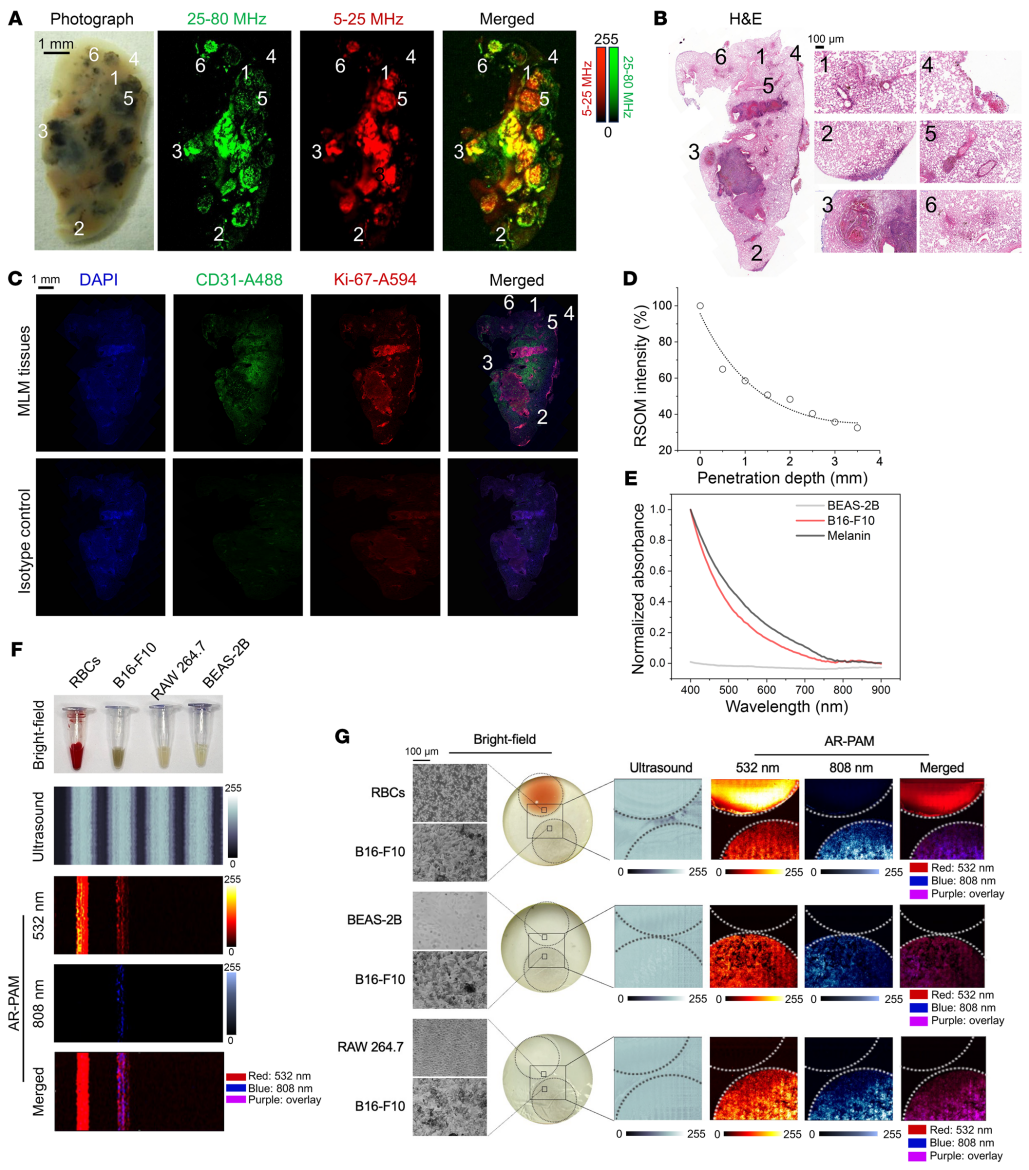
PAI of MLM at mesoscopic and microscopic scales by RSOM and AR-PAM. (**A**) RSOM images of an MLM lobe with US frequencies split into sub-bands that dictate melanoma-associated major (5–25 MHz low frequencies; red) and minor (25–80 MHz high frequencies; green) vascular structures. (**B**) H&E and (**C**) immunofluorescent staining of lung tissue sections from **A**. Magnified histology views of regions of interest are labeled 1–6. (**D**) RSOM intensities of 2 × 10^4^ B16-F10 cells/μL in 2% agarose gel phantoms as a function of the imaging depth. (**E**) Normalized absorption spectra of synthetic melanin, melanogenic B16-F10, and nonmelanogenic BEAS-2B cell suspensions. (**F**) AR-PAM capillary phantom images and (**G**) high-resolution cellular images of RBCs, B16-F10, RAW 264.7, and BEAS-2B cells at 532 and 808 nm. US and phase-contrast bright-field images are included as reference frames.

Next, the microstructural architecture of MLMs was further elucidated using raster-scan optoacoustic mesoscopy (RSOM) functional imaging. Dense raster-scanned US signals were separated into low (5–25 MHz) and high (25–80 MHz) frequency bands, representing large and branched small vascular structures (pseudocolored in red and green, respectively) (Figure 4A). In agreement with histology, smaller lesions showed small, immature structures (subregions 2 and 6) (Figure 4B). In contrast, fast-growing intermediate lesions were primarily characterized by large, hyperpermeable tumor-feeding vasculature with sporadic, developing small vessels (subregions 1 and 5). The tumor-associated hemorrhage, rich in RBCs, angiogenic growth factors, and Hb, confers cancer progression and therapeutic resistance (42, 43). In large and necrotic MLM lesions (subregion 3), a mixed frequency of intricate blood vessel hierarchies with varying diameters and shapes (overlapped in yellow (4A) was detected, corresponding to discontinuous blood supplies and a hypoxic tendency, typical of advanced tumors. Immunofluorescence further validated that proliferative (Ki-67 stain) and high-cellularity (DAPI stain) tumor regions (overlapped in magenta in Figure 4C) exhibited low CD31 expression. However, we noted low cellularity, but histologically positive microsatellite lesions (subregion 4) were overlooked. Due to the visible excitation, the RSOM penetration depth was limited to 3.5 mm (Figure 4D).

### High-resolution AR-PAM imaging of MLMs at the microscale.

**Figure 5:**
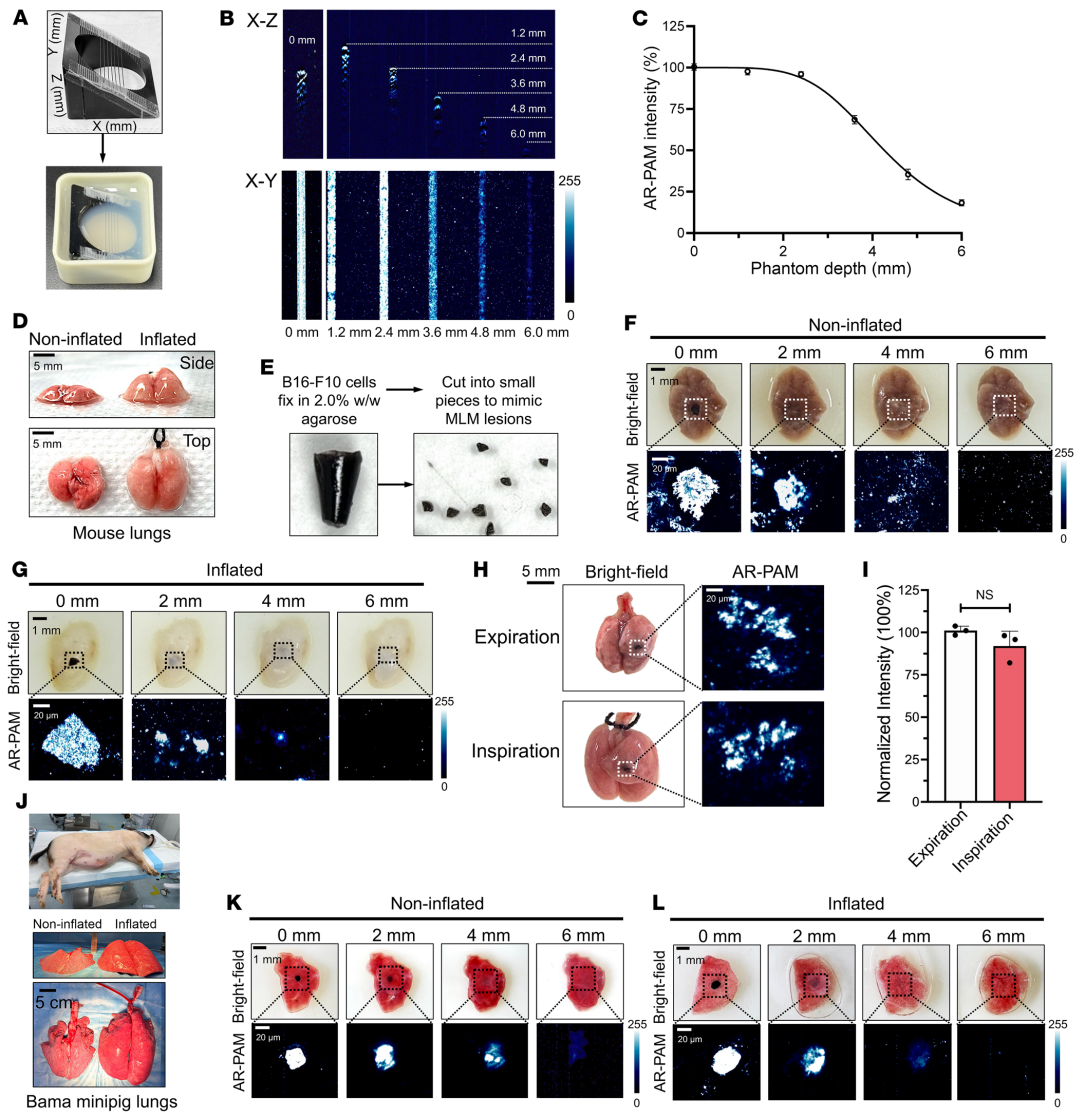
Intraoperative AR-PAM imaging of MLM lesions in inflated lungs of mice and Bama minipigs. (**A**) Photographs of capillary imaging phantoms containing 2 × 10^4^ B16-F10 cells/μL embedded in agarose gels. (**B**) AR-PAM images of capillary phantoms in *x*-*z* and *x*-*y* cross-sections at increasing depths. (**C**) Correlation between the AR-PAM signal intensity and the phantom depth. (**D**) Photographs of noninflated and inflated mouse lungs. (**E**) The process of making a melanoma lesion mimicry. AR-PAM images of a mimetic melanoma lesion implanted in (**F**) noninflated and (**G**) inflated mouse lungs at various depths. (**H**) AR-PAM imaging and (**I**) signal intensities of a melanoma lesion mimicry at 1 mm depth in the same lungs at expiration and inspiration. No statistical significance from the *t* test was found. (**J**) Photographs of noninflated and inflated lungs of Bama minipigs. AR-PAM images of a mimetic melanoma lesion implanted in (**K**) noninflated and (**L**) inflated porcine lung tissues at various depths.

To reach microscopic resolution, we applied AR-PAM imaging in conjunction with anatomic US as a reference modality. B16-F10 melanoma cells displayed higher absorption than amelanotic BEAS-2B epithelial cells across the entire visible-NIR spectrum, emulating a melanin-comparable pattern (Figure 4E). Although the optical profile of melanoma cells allows PA differentiation from RAW 264.7 macrophages and BEAS-2B normal lung cells at 532 nm in an equivalent US background, RBCs with densely packed Hb also underpin strong interfering acoustic signals in response to a light pulse (Figure 4F). Thanks to the sustained absorption of B16-F10 extended into the NIR region, dual positive signals at 532 and 808 nm can specifically resolve melanoma cells at the microscopic resolution (Figure 4G). AR-PAM at 808 nm showed an appreciable imaging depth of 6 mm in phantoms (Figure 5, A–C), exceeding the reported average thicknesses of peripheral and central cadaveric human lungs at 4.1 and 5.9 mm, respectively (44). Unlike MSOT, which includes built-in respiratory and motion compensation algorithms, AR-PAM, with tissue-confined penetration, was studied to image an artificial MLM lesion mimicry during breathing cycles (Figure 5, D and E). The visualization of MLM lesions was attenuated in inflated mouse lungs, yet they still were visible at 4 mm depth (Figure 5, F and G). In the same lungs during inspiration/expiration cycles, no significant PAI signal decay was observed (Figure 5, H and I). Lastly, we achieved a similar detection depth of 4 mm in inflated lungs of Bama minipigs (Figure 5, J–L).

**Figure 6:**
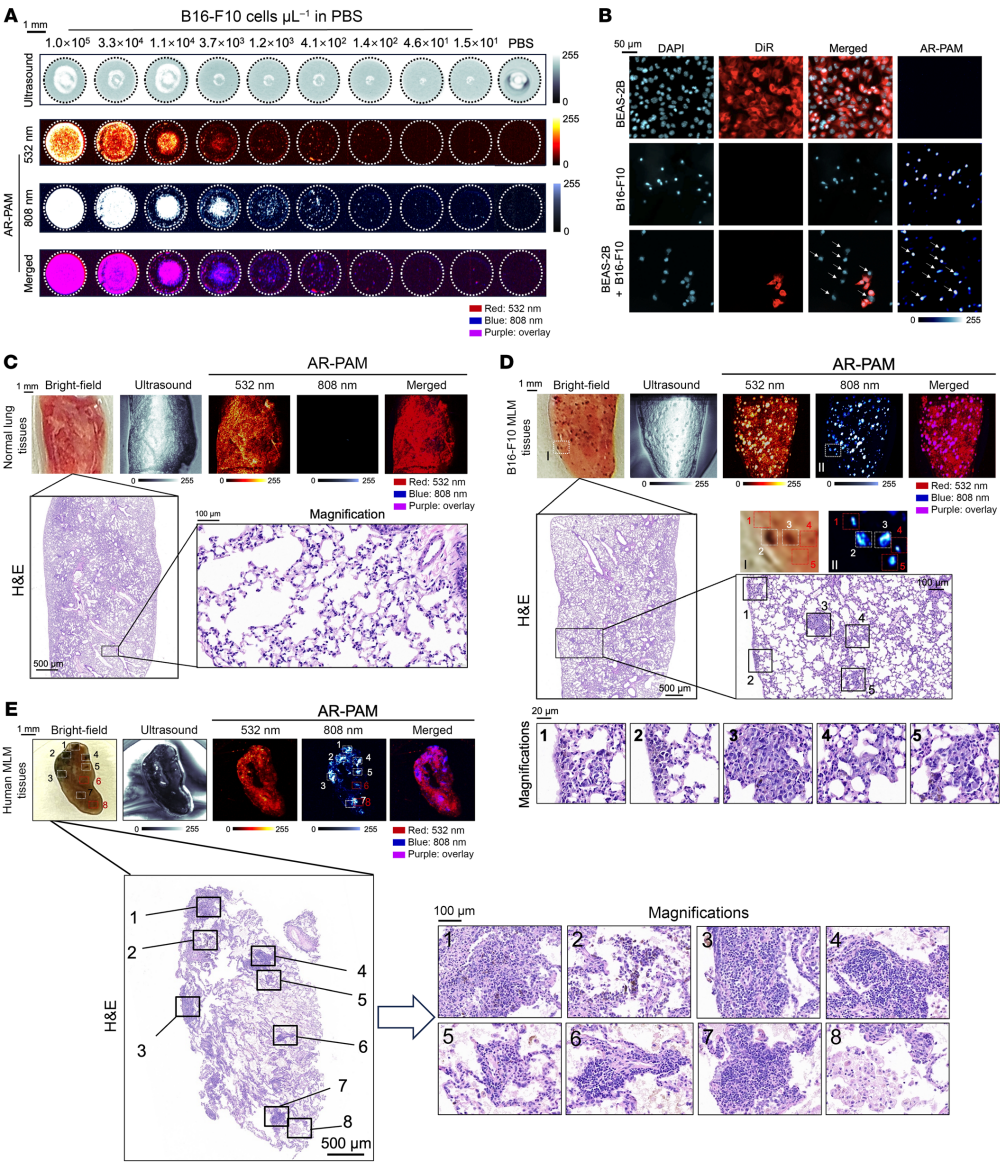
High-resolution AR-PAM imaging of pulmonary metastatic microlesions in MLM mouse models and surgically resected human lung tissues. (**A**) AR-PAM images of B16-F10 cell phantoms at gradient concentrations at 532 and 808 nm dual wavelengths. (**B**) Fluorescence images of PAI-contrasted unstained B16-F10 and 1,1′-dioctadecyl-3,3,3′,3′-tetramethylindotricarbocyanine–labeled BEAS-2B cells. All cells were counterstained with DAPI. AR-PAM images of (**C**) healthy and (**D**) MLM lung tissues resected from mice. (**E**) AR-PAM imaging of patient-derived MLM lung tissues dissected from the operating room. Focused US images are referenced for anatomical registration. Enlarged regions of interest by H&E staining are labeled as indicated and related to photographs and AR-PAM images.

AR-PAM can image an extremely low threshold of approximately 38.3 cells/μL with a linear concentration dependency (*R*^2^ = 0.980) (Figure 6A and Supplemental Figure 2). Moreover, individual B16-F10 cells were readily distinguishable from lung epithelial cells within the mixture at single-cell resolution (Figure 6B). In the pulmonary tissues of healthy mice, PA images were dominated by 532 nm signals, with uniform attenuation and negligible contrast at 808 nm, outlining vascular markings with no focal nodules (Figure 6C). In contrast, lung malignancies in MLM allograft mice were predominantly focal, solitary pulmonary nodules in ill-defined round and oval shapes with spiculated and irregular margins, showing contrast at both wavelengths (Figure 6D and Supplemental Figures 3 and 4). It is worth noting that low-cellularity microlesions with only a few tens of melanoma cells (e.g., subregions 1, 4, and 5 in Figure 6D) can be explicitly detected under AR-PAM, which might otherwise be unidentified under white light.

Having refined the imaging protocol in mouse MLM models, we next wanted to extend its applicability to human lung specimens with MLMs. Concordant with observations in murine models, all pathologically confirmed MLM lesions were accurately demarcated from surrounding normal lung tissue in surgically collected specimens from 3 patients (Figure 6E and Supplemental Figures 5 and 6). Of note, suspicious microlesions were also unobservable under bright-field microscopy (e.g., subregions 6 and 8 in Figure 6E).

### Therapeutic monitoring of low-dose NIR-II photoablation by PAI.

**Figure 7:**
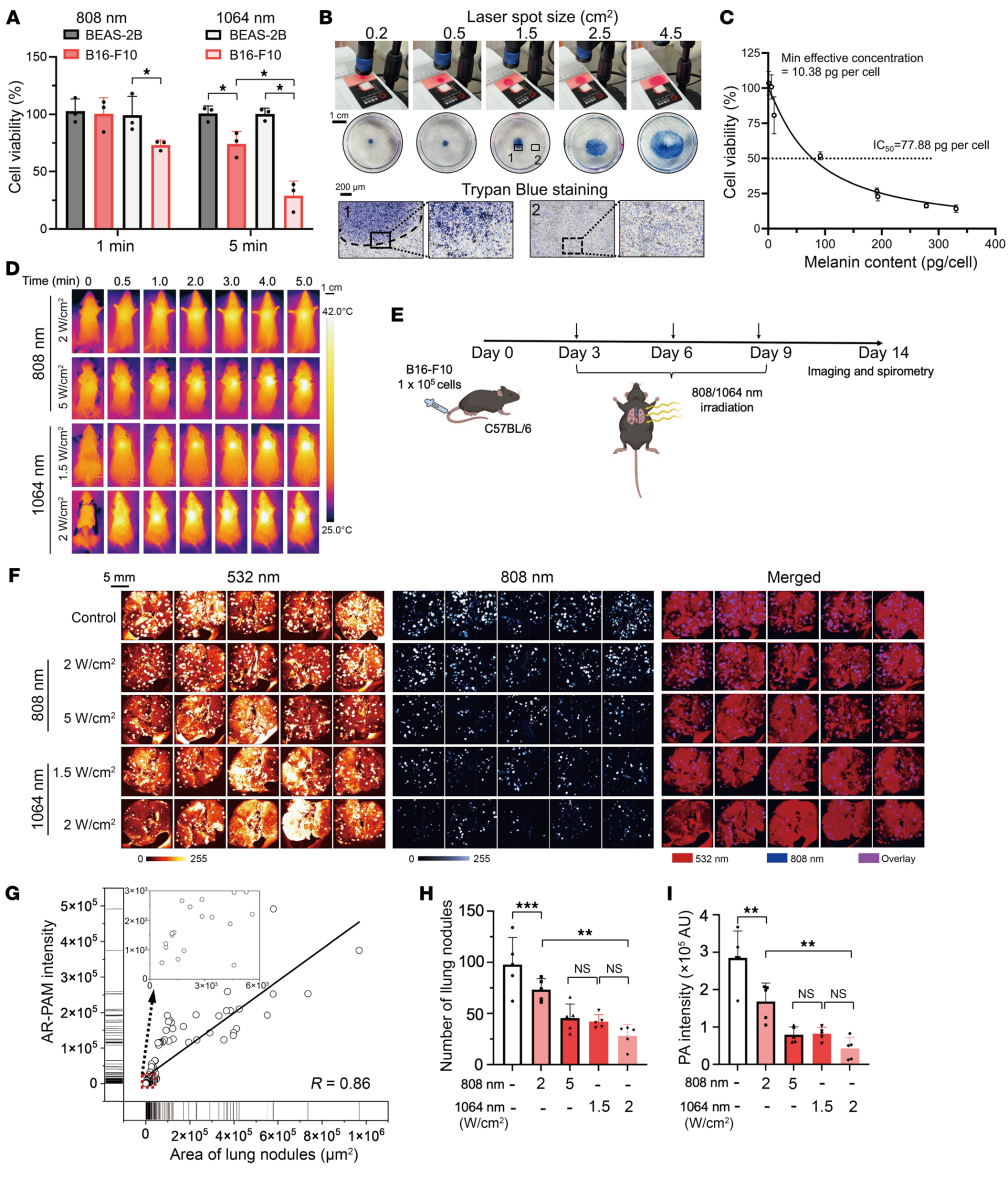
Therapeutic monitoring of drug-free NIR-II photothermal ablation by high-sensitivity AR-PAM imaging. (**A**) Viability of BEAS-2B and B16-F10 cells upon 808 or 1064 nm irradiation for the indicated durations. *P < 0.05 by 1-way ANOVA followed by Tukey’s test. (**B**) Photoablation efficacies at various laser spot sizes. The output laser power was kept constant at 1.5 W. Dead-cell zones from photoablation were detected by trypan blue staining under phase-contrast bright-field microscopy. (**C**) Cell viability of B16-F10 as a function of melanin content after 2 W/cm^2^ photoablation for 5 minutes at 1,064 nm. (**D**) Time-lapse thermographic imaging of B16-F10 MLM mice models receiving NIR-I or NIR-II photoablation at indicated power densities for various durations. (**E**) Therapeutic planning and imaging verification of allograft mice bearing B16-F10 MLM tumor burdens. (**F**) AR-PAM images of lungs harvested from MLM allograft mice at the endpoint after the indicated ablation. Corresponding photographic and US images are in Supplemental Figure 13. (**G**) The correlation between the AR-PAM intensity and the area of MLM nodules (*R* = 0.86). The inset highlights the smallest detected lung nodules. (**H**) The number of MLM nodules in mice receiving different photothermal ablations. (**I**) Quantified PA intensities at 808 nm. **P < 0.01, ***P < 0.001 by 1-way ANOVA followed by Tukey’s test. NS, *P* > 0.05.

Because photothermal ablation can leverage a single laser source for dual use, seamlessly integrated with PAI as adaptive therapy, we first assessed its label-free therapeutic feasibility in MLM mouse models. Capitalizing on dynamic thermographic imaging, we compared NIR-I and NIR-II photoablation modalities at equal irradiation power. B16-F10 cells dynamically escalated thermal elevation more rapidly at both wavelengths than did BEAS-2B cells and culture media, resulting in higher localized temperatures with NIR-II (Supplemental Figure 7). Cell viability assays also demonstrated higher in vitro cytotoxicity against melanoma cells at NIR-II, with a rapid onset (~1 minute) and minimal off-target effects on BEAS-2B cells (Figure 7A). Meanwhile, the ablation area could be precisely controlled by the laser spot size to induce cell death (Figure 7B and Supplemental Figure 8). The photoablation was likewise governed by melanogenesis, which was inhibited by 5-chloro-2-mercaptobenzimidazole (tyrosinase-IN-22) in a dose-dependent manner (Supplemental Figure 9). The minimal effective intracellular melanin concentration per cell was determined to be 10.38 pg with an IC_50_ of 77.88 pg (Figure 7C).

Despite the deeper tissue penetration of NIR-II, water molecules ubiquitous in the body also exhibit strong intrinsic absorption at NIR-II, leading to nonspecific overheating of surrounding healthy tissues during procedures and necessitating an optimal dose. Although more penetrative NIR-II ablation was still modestly dampened in inflated lungs at up to a 6 mm depth (Supplemental Figure 10), ablative energy was delivered more efficiently at NIR-II (1.5 W/cm^2^) than at NIR-I (5 W/cm^2^) to reach a comparable temperature in living mice with MLMs (Figure 7D and Supplemental Figure 11). Moreover, the low induction dose was relatively safe in vivo because the ablative temperature in the lungs was significantly higher in MLM mice than in healthy counterparts, due to perturbed melanogenesis (Supplemental Figure 12).

Next, we explored PAI-guided monitoring of photoablation in mice with MLMs (Figure 7E). In line with in vitro findings, NIR-II photoablation yielded a comparable therapeutic efficacy at a lower power (1.5 vs. 5 W/cm^2^) or an improved outcome at an equivalent power (2 W/cm^2^) in comparison with conventional NIR-I, as evidenced by the number of lung nodules (Figure 7F and Supplemental Figure 13). High-resolution AR-PAM imaging with a US anatomic reference not only identified the localization and contours of MLM lung nodules down to as small as 658.3 μm^2^ but also gauged the tumor extent in measurable correlation (*R* = 0.86) with PAI intensities (Figure 7, G–I). Furthermore, we observed that even low-dose NIR-II photoablation exhibited therapeutic efficacy and extended survival in mice with advanced-stage MLM tumors (twice the tumor inoculation amount and growth time), whereas NIR-I produced no benefit (Supplemental Figure 14).

### Physiological evaluation of respiratory functions by whole-body plethysmography.

**Figure 8:**
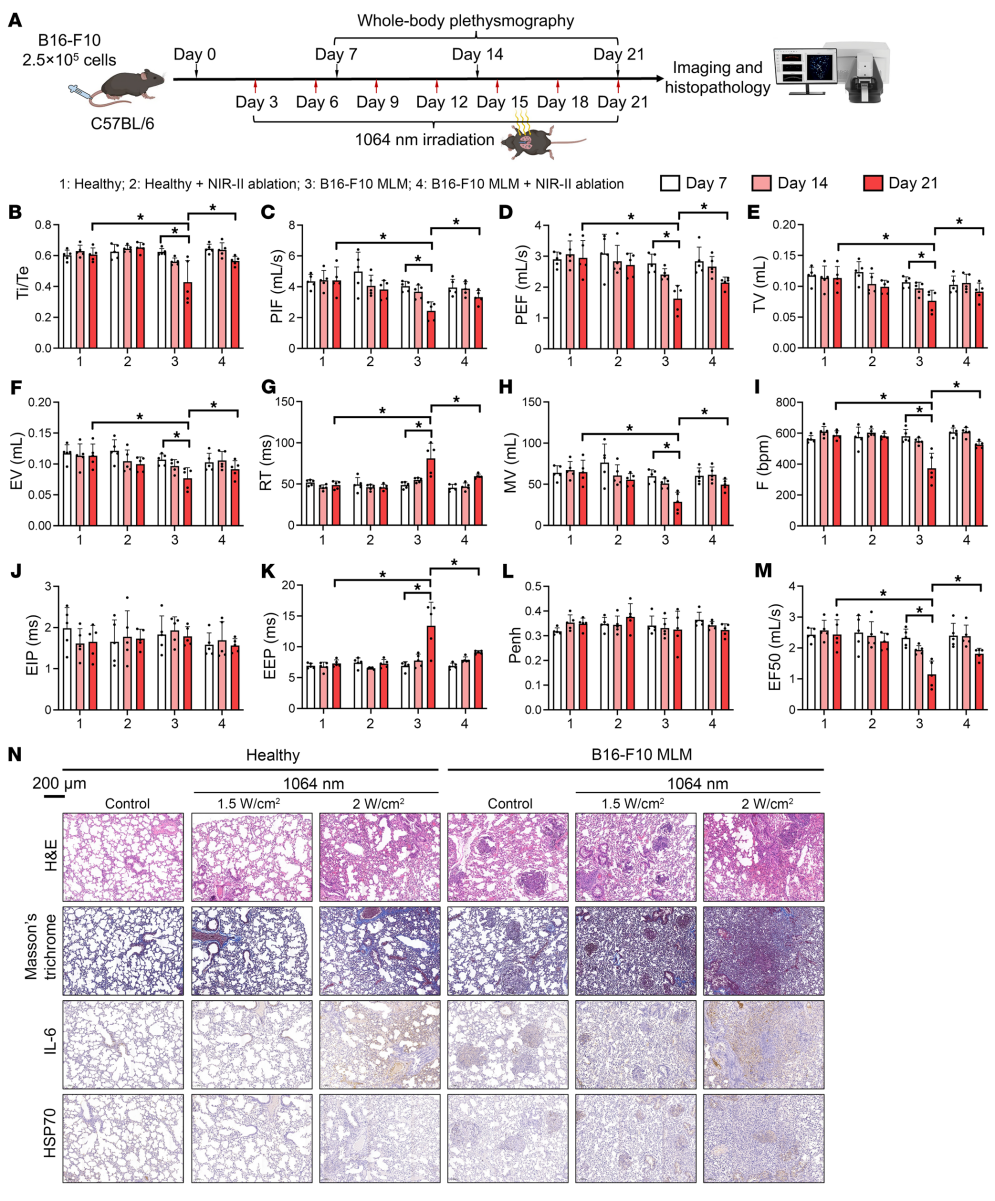
Evaluation of pulmonary functions by longitudinal WBP and histology after NIR-II photoablation in the healthy and B16-F10 MLM mice. (**A**) Schematic diagram showing the planning of NIR-II ablation therapy (1.5 W/cm^2^ for 5 minutes per cycle of treatment), WBP, imaging, and histopathology for allograft mice bearing MLMs. Group assignments are indicated by numbers 1 to 4 (*n* = 5). WBP indices on days 7, 14, and 21 include (**B**) the ratio of inspiratory time to expiratory time (TI/TE), (**C**) peak inspiratory flow rate (PIF), (**D**) peak expiratory flow rate (PEF), (**E**) tidal volume (TV), (**F**) expiratory volume (EV), (**G**) relaxation time (RT), (**H**) minute ventilation volume (MV), (**I**) respiratory rate (abbreviated F), (**J**) end-inspiratory pause (EIP), (**K**) end-expiratory pause (EEP), (**L**) bronchoconstriction coefficient (Penh), and (**M**) forced expiratory flow at 50% vital capacity (EF50) of healthy and MLM allograft mice upon photoablative interventions. *P < 0.05 by 1-way ANOVA followed by Tukey’s test. (**N**) H&E, Masson’s trichrome, and IHC staining of lung tissues resected from healthy and B16-F10 MLM allograft mice on day 14 after photoablation.

Whole-body plethysmography (WBP) complements diagnostic imaging and provides quantitative measures of alterations in airflow limitation, hyperinflation, or reduced lung compliance that may arise from photoablation and tumor-induced obstruction. In healthy mice, NIR-I ablation was well tolerated, with no adverse effects observed at either dose (Supplemental Figure 15). By contrast, NIR-II only affected the ratio of inspiratory time to expiratory time and the relaxation time at the high delivered dose (Figure 8, A–M, and Supplemental Figure 15). Lung mechanical indices were also evaluated in MLM mouse models that received either NIR-I or NIR-II photoablation. The progression of the MLM tumor load significantly decreased several pulmonary function indices that had been aggravated over time (Figure 8, A–M). Both NIR-I and NIR-II ablation procedures effectively restored MLM-deteriorated pulmonary function (Supplemental Figure 16). However, despite its higher therapeutic efficacy (Figure 7, H and I), overdosing with NIR-II was less effective at restoring lung compliance in healthy mice, due to respiratory side effects. In contrast, optimally deposited NIR-II recovered MLM-impaired lung functions without observable adverse effects (Figure 8, A–M). The conclusion is further supported by Masson’s trichrome staining and IHC, which revealed lower collagen, IL-6, and HSP70 levels at the optimal therapeutic dose (Figure 8N).

## Discussion

Spatiotemporally complementary imaging, exemplified by PET-CT and PAI-US, ordinarily relies on different modalities (45). Meanwhile, planar x-ray imaging, in addition to conventional in vivo scans such as radiography, fluoroscopy, and densitometry, has been used to scrutinize the structure and physiology of live cells and organs at high resolution (46, 47). Nevertheless, fluorescence imaging variants spanning fluorescence molecular tomography, widefield mesoscopy, and high-resolution microscopy are appreciated to overcome the penetration and diffusion limits of individual techniques for imaging living animals, resected organs, and histology sections with the same signal source of probes in preoperative, intraoperative, and postoperative scenarios over diverse scales (48, 49). With equal total pixels, finer features can be resolved with a smaller pixel size, whereas a larger field of view (FOV) with fewer pixels yields lower-resolution images. Needle-based NIR confocal laser endomicroscopy with 3 probes spanning FOVs of 600, 325, and 240 μm was developed to image cancer cells at different lateral resolutions (50). Akin to fluorescence imaging, PAI may also entail trade-offs between FOV and spatial resolution. Ideally, the integrated multiscale imaging workflow would require no specific labeling to report the presence of MLM masses within a large FOV and to identify multiregional microsatellite lesions comprising only tens of cancer cells at high spatiotemporal resolution (Figure 1).

In our study, we explored the PAI principle, which stems from sustained dysregulation of melanogenesis signaling in melanoma cells (41). MLM lung tissues are significantly heavier than healthy lungs (Figure 3B). The phenomenon is attributed not only to the tumor burden but also to interrelated factors, including cancer edema, angiogenesis, tumor-associated inflammation, and desmoplastic progression, which lead to elevated deposition of cancer cells, infiltrating immune cells, blood cells, and stromal cells. Therefore, imaging at higher resolution beyond the whole-body scale is required to reveal tumor microenvironmental information and to avoid omitting microlesions. At the macroscale, MSOT detects the body-wide presence of MLMs in the lungs noninvasively. Moving to the mesoscale, RSOM with separate US frequency bands enables the characterization of tumor vasculature, bleeding, and necrosis. Microscopically, AR-PAM depicts tumor margins with high contrast and histopathological resolution, revealing detailed cellular and nodular features. Such a streamlined, operationally convenient approach enables visualization of complex MLM processes, assessment of tumor burden and distribution, and tracking of NIR-II therapeutic efficacy.

MLM lesions disperse throughout the lungs in a diffuse pattern (Figure 6D), making complete surgical excision particularly challenging. As a noncontact, minimally invasive intervention for eradicating tumor masses via focal heating, NIR light-enabled photothermal ablation has been preliminarily explored in clinical studies (51). On the other hand, label-free photoablation has long been used in clinics to remove benign pigmented nevi and birthmarks with uneventful recovery. Given the potential to share a single laser source, integrating real-time PA feedback into minimally invasive photoablation allows clinicians to confirm that the intended treatment zone is positive while sparing surrounding healthy tissues and minimizing collateral damage (Supplemental Figure 17). The capacity to correlate localized ablation with PA patterns converges diagnostic imaging and minimally invasive therapy, enabling an instant switch between “imaging mode” and “therapeutic mode” and enormously enhancing treatment accuracy. PA image guidance would facilitate patient-specific dose modulation and timing, representing a leap forward in precision cancer intervention. Nonetheless, ongoing challenges persist. For instance, despite NIR-II’s superior ablation efficacy compared with NIR-I (Figure 7), strong water absorption in the spectral window may cause unintended thermal damage at high delivered doses (Supplemental Figure 15), compromising therapeutic outcomes. Several strategies can be used to resolve the problem: (a) exogenous targeted agents with strong NIR-II absorption that preferentially accumulate in MLM lesions and concentrate energy to lower required doses; (b) optimization of laser pulse duration, power, and treatment courses; (c) advanced laser delivery systems such as fiber optic or thoracoscopic probes to better direct energy deposition with minimal dispersion; and (d) cooling procedures for patient preparation like cryospray and ice water recycling under thermometric surveillance. Future work should also focus on hardware integration of multiscale PAI and photoablation, especially with an emphasis on incorporating emerging NIR-II PAI (52). Finally, algorithms are desired to compensate for respiratory motion artifacts to minimize blurring and misregistration. NIR-II photoablation may be deployed as an alternative, adjunct, or salvage ablation modality to clinically established energy-based techniques like high-intensity focused US, irreversible electroporation, radiofrequency ablation, and microwave ablation to balance depth-precision-safety trade-offs, particularly for technically challenging lesions or residual or recurrent disease. With precise melanin manipulation even in amelanotic cancer (53), the streamlined theranostic approach may be extended to other superficial malignancies, such as cutaneous metastases, head and neck cancer, breast cancer, and near-surface abdominal lesions.

### Conclusion.

On a label-free basis, we present a hierarchical theranostic workflow that leverages the strengths of tomographic MSOT, mesoscopic RSOM, and histology-grade AR-PAM to comprehensively evaluate MLM cancerous lesions. Shifting photoablation from NIR-I to NIR-II not only provides deeper tissue penetration but also improves therapeutic outcomes. Melanoma is considered the most immunogenic neoplasm for its high DNA mutational burden. Photoablation can further trigger neoantigen release to empower immunotherapy (54–56). By progressively enhancing spatial resolution and focusing on areas of concern, sequential imaging can facilitate timely detection, accurate diagnosis, and informed therapy decisions. The ease of PAI integration with established US allows for seamless adaptation in clinical practice, and high-resolution AR-PAM may provide corroborative evidence for clinical histopathology. The combination of structural, functional, and molecular information, when streamlined, holds promise for advancing the imaging and guided therapy of MLM in clinical settings.

## Methods

### Sex as a biological variable.

All mouse and Bama minipig models were female in this study. Human MLM samples were collected from both sexes (*n* = 2 male and 1 female). Sex was not considered a biological variable for imaging of preclinical models and human samples. It is unknown whether sex affects ablation efficacy.

### Bioinformatic analysis.

Normalized RNA-Seq data of primary melanoma (*n* = 103), melanoma cutaneous (*n* = 8) and lung metastases (*n* = 8), and peritumoral normal tissues of LUAD (*n* = 62) and LUSC (*n* = 51) were retrieved from the Genomic Data Commons (GDC) portal of The Cancer Genome Atlas (TCGA) database. Gene expression data of nonmelanocytic BCC (*n* = 15) and normal skin tissues (*n* = 6) were accessed via GSE269601 in the Gene Expression Omnibus (GEO) database (57). Averaged transcripts per million counts were extracted, processed, and normalized by *z* scores, with heatmaps generated based on the melanogenesis gene signature from the Kyoto Encyclopedia of Genes and Genomes, using the R package ComplexHeatmap, version 2.20.0, in R, version 4.4.1 (58). Batch-corrected expression data of melanoma cell lines were sourced from the Cancer Cell Line Encyclopedia through the DepMap portal. Additionally, the expression profiles of normal human epidermal keratinocytes were accessed via GSE66412 from the NCBI GEO (59). A 2-tailed Student’s *t* test was performed to identify differential gene expression with a *P* value < 0.05 between melanoma and normal epidermal keratinocytes within the melanogenesis gene signature. Selected differentially expressed genes were mapped based on *z*-scored transcripts per million values. To remove batch effects arising from different data sources, we applied correction algorithms using the combat function from the R package Sva, version 3.52.0 (60).

### Cell lines.

All cell lines were obtained from the American Type Culture Collection. B16-F10 murine melanoma cells and BEAS-2B human normal lung epithelial cells were cultured in DMEM (HyClone) supplemented with 10% fetal bovine serum (Gibco) and 1% penicillin-streptomycin (Gibco) in a 5% CO_2_ humidified atmosphere at 37°C. RAW 264.7 mouse macrophages were cultured in RPMI 1640 medium (HyClone). Cell numbers were automatically quantified in PD100 counting chambers using a Cellometer Auto 1000 counter (Nexcelom Bioscience) after trypan blue staining. B16-F10 cells were transduced with the GFP-luciferase (Luc) reporter system via lentivirus vectors, as we previously described (13, 61).

### Animal models.

J:NU outbred, athymic, female nude mice 4–6 weeks old were acquired from The Jackson Laboratory for MSOT and RSOM without additional shaving. We obtained 6-week-old wild-type C57BL/6 female mice from the Guangdong Medical Laboratory Animal Center, and these were used in all other studies, including AR-PAM, photoablation therapy, and spirometry. All animals were housed in a pathogen-free environment (22°C, 12-hour light/12-hour dark cycle, 40%–50% humidity) and quarantined for at least 1 week for acclimatization with unrestricted access to food and water and periodic health inspections. MLM mouse models were established by intravenously injecting 1 × 10^5^ to 2 × 10^5^ B16-F10 or B16-F10-Luc melanoma cells in PBS through the tail vein of mice (*n* = 3 for imaging and 5 for therapy in each group at a minimum, according to animal research’s 3Rs principle (62). Tumor growth patterns were tracked periodically by BLI after intraperitoneal injection of 150 mg of d-luciferin (Cayman Chemical) per kg body weight. Mice were randomly assigned to experimental groups to receive procedures without any other randomization or blinding procedures in place. Female Bama minipigs aged 4–6 months (*n* = 1 in each group) were procured from Guangzhou Huateng Biomedical Technology as porcine models.

### Lung inflation of mice and Bama minipigs.

Mice and Bama minipigs were euthanized according to procedures approved by the IACUC of Huateng Biomedical Technology Co. Following thoracotomy, the rib cage was removed to allow clear access to the lungs. The connective tissue surrounding the trachea was carefully dissected. A syringe needle was then inserted into the tracheal lumen while maintaining the needle parallel to the airway (63). The lungs were slowly perfused and inflated with 10% neutral buffered formalin until fully expanded, which was confirmed by a slight return flow of fixative. The trachea was subsequently secured by placing a suture around it (tied initially over the needle, then tightened after needle withdrawal) to maintain inflation. Artificial MLM lesions were made by fixing 2 × 10^7^/mL B16-F10 cells in 2.0% wt/wt agarose, cutting them into small pieces, and embedding them into inflated or noninflated lungs for imaging.

### Collection of human MLM surgical tissues.

Human lung tissues were surgically excised from patients with cancer with a confirmatory diagnosis of melanoma pulmonary metastases between 2014 and 2024 at the Sun Yat-sen University Cancer Center.

### Characterization of melanin in B16-F10 cells.

B16-F10 cells were seeded in 6-well plates and incubated for 48 hours. We collected and solubilized 1 × 10^6^ cells in 100 μL of a 1 M NaOH/10% DMSO (vol/vol) mixture at 80°C for 2 hours. The UV-vis-NIR spectrum of B16-F10 cells was recorded using a UV-2600 spectrophotometer (Shimadzu) and compared with those of synthetic melanin (Sigma) and nonmelanogenic BEAS-2B normal lung epithelial cells. Anti-melanogenesis was induced by adding tyrosinase-IN-22 (MCE) to cultured B16-F10 cells in 12-well plates for 24 hours of incubation to inhibit tyrosinase substrates (l-tyrosine and l-DOPA). Next, 5 × 10^5^ cells were collected and solubilized in 100 μL of a 1 M NaOH/10% DMSO (vol/vol) mixture at 80°C for 2 hours. The melanin content was determined by measuring absorbance at 475 nm with reference to a standard calibration curve of synthetic melanin.

### Imaging setup.

Macroscopic whole-body MSOT imaging was conducted using a preclinical inVision 256-TF MSOT system (iThera Medical), as previously reported (13, 61). The system used a Q-switched Nd:YAG laser for optical excitation (pulse duration ≤10 ns, repetition rate 10 Hz) and a cylindrically focused 256-element transducer array for US detection (5 MHz central frequency with up to 270° coverage). The imaging plane was set to align with the 10-armed illumination fiber bundle. Data were acquired in a water bath at a constant temperature of 34°C for optimal coupling. Animals were topically applied with US couplant gels and wrapped in a thin polyethylene film to enhance coupling and suppress breathing motion under 2% isoflurane anesthesia. Scans were set from 680 to 900 nm in 10 nm steps at 3 frames per wavelength to generate 2D traverse-plane images. The maximal penetration depth was evaluated using a clinical-grade handheld MSOT Acuity probe (iThera Medical). Images were reconstructed using back-projection algorithms with the 50 kHz–6.5 MHz finite impulse response data filter in the ViewMSOT software, version 4.0.3.4. The data were then linearly unmixed pixel by pixel, and negative values were discarded. The melanin-specific optoacoustic spectrum was derived from factory presets and differentiated from intrinsic biological absorbers, such as HbO_2_ and Hb.

Mesoscaled RSOM was performed in a 34°C water bath on a prototype scanner (iThera Medical) under nanosecond-pulsed laser epi-illumination at 532 nm (1 ns, 2 kHz, and 1 mJ) using a 3-arm fiber bundle, as previously described (64). Optoacoustic signals were collected using a spherically focused detector with a central frequency of 50 MHz and a bandwidth spanning 5–80 MHz, which were further amplified by a low-noise amplifier with a gain of 63 dB. Signals from continuous-discrete scanning at a raster step size of 20 μm were split into 2 frequency sub-bands — low frequencies (5–25 MHz) and high frequencies (25–80 MHz) — to visualize large and small lung structures in reconstructed color codes. To improve RSOM image quality, blood was removed from fresh lung tissues by repetitive washing with PBS.

Microscopic AR-PAM imaging used dual-pulsed laser excitations at 532 nm (HLX-G, Leukos; pulse width: 1 ns) and 808 nm (AOML-150B, Nanjing Institute of Advanced Laser Technology; pulse width: 8 ns) on a PASONO-ANI small-animal imaging system with PAI-US dual modalities (Guangdong Photoacoustic Technology). Pulsed US modules (±50 V; single cycle per pulse; central frequency: 30 MHz; 6 dB bandwidth: 100%; pulse width: 32 ns) were triggered at a repetitive frequency of 10 kHz via a field-programmable gate array. The laser beam was focused through a 4× objective lens, and a self-focusing ultrasonic transducer (central frequency: 30 MHz; 6 dB bandwidth: 80%; focal length: 8 mm; outer diameter: 8 mm; center hole diameter: 3 mm) was submerged in water, with the imaging window sealed with a transparent thin film. The laser and US focal points were aligned for optimal PAI detection efficiency, enabling high-resolution imaging of melanoma distribution in mouse lungs. Cells were imaged in glass capillaries or on round coverslips. PAI images were displayed with intensity quantitatively analyzed in ImageJ. For microscopic single-cell visualization, B16-F10 cells were mixed at a 1:2 ratio and co-cultured for 6 hours with BEAS-2B cells, which were prelabeled by the lipophilic membrane dye 1,1′-dioctadecyl-3,3,3′,3′-tetramethylindotricarbocyanine iodide (MeilunBio), and then subjected to AR-PAM. The same slides were then mounted in the mounting medium containing DAPI for coregistered fluorescence imaging under an identical FOV using the KF-FL-400 Digital Pathology Slide Scanner (KFBIO).

### In vitro photothermal characterization.

We seeded 2.5 × 10^3^ B16-F10 cells per well in 96-well plates. After 24 hours of incubation, cells were irradiated with a NIR-I laser at 808 nm or a NIR-II laser at 1,064 nm at a power density of 2 W/cm^2^ for 5 minutes, monitored in real time by thermographic imaging with a FLIR ONE Pro camera. Temperatures were quantified from imaging using the FLIR Tools Thermal Analysis software. The empty medium and nonmelanoma BEAS-2B cells were included as controls. Following irradiation, cells were further incubated for an additional 24 hours, with media replaced with fresh media containing 0.5 mg/mL MTT. After 4 hours, 150 μL of DMSO was added to dissolve the formazan crystals, and absorbance was measured at 490 nm using an Infinite M200 Pro multimode microplate reader (Tecan). Absorbance at 630 nm was measured simultaneously as a reference. Live/dead staining was performed after NIR-II irradiation using a Calcein-AM/propidium iodide (PI) Double Stain Kit (Yeasen Biotech) according to the manufacturer’s instructions to label intracellular esterase of live cells and nuclei of membrane-compromised dead cells. Fluorescence images were taken at excitation/emission of 494/517 nm for live cells and at 535 nm/617 nm for dead cells, respectively. The influence of laser spot size was visualized using infrared viewing cards (Daheng Optics) and evaluated by staining with a 1:1 mixture of 0.4% (wt/vol) trypan blue and DMEM to delineate ablation boundaries.

### Photothermal ablation therapy in living mice with MLMs.

Female C57BL/6 mice with MLM burdens were randomly assigned into 5 groups (*n* = 5 each) for therapeutic assessment: no interventions, 808 nm NIR-I ablation (2.0 or 5.0 W/cm^2^), and 1,064 nm NIR-II ablation (1.5 or 2.0 W/cm^2^). NIR-I and NIR-II ablative interventions were performed at equivalent power densities or temperatures, consisting of three 5-minute sessions, each administered every 3 days. Prior to photothermal ablation, fur was removed from each mouse’s neck and upper chest using electric clippers and depilatory cream to minimize interference and optimize therapeutic results. At the endpoints, mice were sacrificed, and their lung tissues were collected for imaging and histological analysis.

### Histology.

Lung tissues were freshly excised, fixed in 4% paraformaldehyde in PBS at 4°C for 24 hours, dehydrated, embedded in paraffin wax, and cut into 2 μm ultrathin sections using an automated microtome. The paraformaldehyde-fixed paraffin-embedded sections were stained with H&E or Masson’s trichrome staining and scanned in the bright field. The Discovery XT processors (Ventana Medical Systems) were used for immunofluorescence. Sections were deparaffinized and conditioned, with antigens retrieved in CC1 buffer (Ventana Medical Systems), blocked in Background Buster (Innovex), and incubated with avidin/biotin blocking reagent (Ventana Medical Systems). Primary antibodies against CD31 (Dianova, DIA-310) and Ki-67 (Abcam, ab16667) were applied at 1:250 and 1:1,000 dilutions for 5 hours, followed by 1:200-matched biotinylated secondary antibody (Vector Labs) staining for 1 hour. At the manufacturer’s recommended dilutions, the detection was performed with Streptavidin-HRP D (DAB Map kit; Ventana Medical Systems) and Tyramide Alexa Fluor 488 and 594 (Invitrogen). Lastly, samples were counterstained with DAPI for 10 minutes and mounted in the Mowiol medium (Calbiochem). IHC was performed at the Guangzhou Huayin Medical Laboratory Center by incubating tissue sections with primary antibodies to HSP70 (Proteintech, 109951-AP) and IL-6 (Affinity, DF6087) at dilutions of 1:200 and 1:50, respectively. 3D histopathology was reconstructed from serial sections stained with H&E, as we reported previously (65). Bright-field scans were conducted using a Pannoramic digital slide scanner (3DHistech). Images were aligned and reconstructed using the Voloom software (microDimensions). Segmentation and color deconvolution of histologically confirmed tumor regions were performed in ImageJ to yield pseudocolored images, with nontumor lung tissue in a separate channel. Aligned stacks were visualized in Imaris (Bitplane) to denote tumor lesions in 3D surfaces.

### WBP.

Conscious mice were placed in unrestrained chambers of a WBP-4A pulmonary function system (EMKA Technologies), which was calibrated before use. The mice were allowed to acclimate for 5 minutes to adapt to the environment and were noninvasively measured for respiratory variables related to pulmonary function in the conscious state.

### Statistics.

Quantitative data are presented as mean ± SEM and analyzed using the unpaired, 2-tailed Student’s *t* test or 1-way ANOVA with Tukey’s test to compare multiple groups in GraphPad Prism. Differences in survival curves were assessed using a 2-sided log-rank test. *P* values of < 0.05, 0.01, 0.001, and 0.0001 (denoted by *, **, ***, and ****, respectively, in figures) were considered statistically significant at various levels.

### Study approval.

All animal experiments were approved by the IACUCs at the Memorial Sloan Kettering Cancer Center (approval 06-07-011) and the Sun Yat-sen University Cancer Center (approval 2024001575) in strict compliance with the NIH *Guide for the Care and Use of Laboratory Animals* (National Academies Press, 2011). All procedures involving Bama minipigs were reviewed and approved by the IACUC of Huateng Biomedical Technology Co. (approval B202509-32). The study protocol (B2024-422-01) for collecting human MLM samples was approved by the Ethics Committee at the Sun Yat-sen University Cancer Center.

### Data availability.

All original data are available in the public open data-sharing database, Research Data Deposit (identifier RDDB2026335422). Sequencing data in the study were from publicly available MINSEQE-compliant repositories. RNA-Seq data of TCGA-SKCM, TCGA-LUAD, and TCGA-LUSC were retrieved from the TCGA GDC portal. BCC and normal skin samples, as well as normal human epidermal keratinocytes, can be assessed from the NCBI GEO database (accession nos. GSE269601 and GSE66412, respectively). Melanoma cell line data are available from DepMap Public Release 24Q2. Data points in all graphs are provided in the Supporting Data Values file.

## Author contributions

JY, JG, and XY conceived and designed the study. JY, KH, KXVP, CA, and QZ carried out MSOT and RSOM imaging experiments. WX, YZ, HW, ZL, RH, and KS performed AR-PAM imaging and ablation therapy. CW and YZ conducted bioinformatic analysis and visualized the data. JY, JG, XY, and MK co-supervised the project and provided resources. JY and WX wrote the manuscript, which was proofread and approved by all authors for submission.The order of co-first authors was determined alphabetically.

## Conflict of interest

The authors have declared that no conflict of interest exists.

## Funding support

This work is supported in part by NIH funding and is subject to the NIH Public Access Policy. Through acceptance of this federal funding, the NIH has been given a right to make the work publicly available in PubMed Central.

- National Natural Science Foundation of China (grants 82472041, 82071978, and 52271196 to JY).
- Guangdong Provincial Joint Fund for Corporate Innovation and Development (grant 2024A1515220058 to JY).
- National Key Research and Development Program of China (grant 2021YFF1200700 to JY).
- Young Talents Program of Sun Yat-sen University Cancer Center (grant YTP-SYSUCC-0024 to JY).
- Fundamental Research Funds for the Central Universities at Sun Yat-sen University (grant 31610026 to JY).
- National Cancer Institute (grant R01 CA212379 to JG).
- NIH Cancer Center Support Grant (grant P30 CA008748 to at Memorial Sloan Kettering Cancer Center Radiology).

## Supplementary Material

Supplemental data

Supporting data values

## Version 1 05/01/2026

Electronic publication

## References

[1] (2024). Trends in melanoma incidence, prevalence, stage at diagnosis, and survival: An analysis of the United States Cancer Statistics (USCS) Database. Cureus.

[2] Orchard GE, Calonje E (1998). The effect of melanin bleaching on immunohistochemical staining in heavily pigmented melanocytic neoplasms. Am J Dermatopathol.

[3] Tas F (2012). Metastatic behavior in melanoma: timing, pattern, survival, and influencing factors. J Oncol.

[4] Liu D (2024). Selective organ-targeting hafnium oxide nanoparticles with multienzyme-mimetic activities attenuate radiation-induced tissue damage. Adv Mater.

[5] Predina JD (2018). Identification of a folate receptor-targeted near-infrared molecular contrast agent to localize pulmonary adenocarcinomas. Mol Ther.

[6] Okusanya OT (2014). Intraoperative near-infrared imaging can identify pulmonary nodules. Ann Thorac Surg.

[7] Azari F (2023). Evaluation of OTL38-generated tumor-to-background ratio in intraoperative molecular imaging-guided lung cancer resections. Mol Imaging Biol.

[8] Bou-Samra P (2023). Cathepsin detection to identify malignant cells during robotic pulmonary resection. Transl Lung Cancer Res.

[9] Okusanya OT (2015). Intraoperative molecular imaging can identify lung adenocarcinomas during pulmonary resection. J Thorac Cardiovasc Surg.

[10] Choi HS (2022). Targeted intraoperative fluorescence imaging for the visualization of ground-glass nodules in the lung. Transl Lung Cancer Res.

[11] Kashiwagi S, Choi HS (2023). Ovarian cancer-targeted near-infrared fluorophores for fluorescence-guided surgery. Ann Transl Med.

[12] Sarkaria IS (2023). Pafolacianine for intraoperative molecular imaging of cancer in the lung: the ELUCIDATE trial. J Thorac Cardiovasc Surg.

[13] Yang J (2021). Structurally symmetric near-infrared fluorophore IRDye78-protein complex enables multimodal cancer imaging. Theranostics.

[14] Ntziachristos V (2025). Addressing unmet clinical need with optoacoustic imaging. Nat Rev Bioeng.

[15] Zhao J (2025). Correcting for sub-resolution tissue motion in localization optoacoustic tomography. Opt Lett.

[16] You Y (2025). High-sensitivity cell-based plasmonic biolayer interferometry with a two-tiered enhancement for clinical autoimmunity surveillance of pemphigus vulgaris. ACS Nano.

[17] Bader M (2025). Low frequency detection in clinical multispectral optoacoustic tomography. Photoacoustics.

[18] Karlas A (2024). Multiscale optoacoustic assessment of skin microvascular reactivity in carotid artery disease. Photoacoustics.

[19] Kalva SK (2025). Toward noninvasive optoacoustic imaging of whole-heart dynamics in mice. Light Sci Appl.

[20] Breathnach A (2018). Preoperative measurement of cutaneous melanoma and nevi thickness with photoacoustic imaging. J Med Imaging (Bellingham).

[21] Kosik I (2019). Intraoperative photoacoustic screening of breast cancer: a new perspective on malignancy visualization and surgical guidance. J Biomed Opt.

[22] Gao S (2023). Intraoperative laparoscopic photoacoustic image guidance system in the da Vinci surgical system. Biomed Opt Express.

[23] Fakhoury JW (2024). Photoacoustic imaging for cutaneous melanoma assessment: a comprehensive review. J Biomed Opt.

[24] Schroeder E (2013). Average chest wall thickness at two anatomic locations in trauma patients. Injury.

[25] Azizi N (2020). Optimal anatomical location for needle chest decompression for tension pneumothorax: a multicenter prospective cohort study. Injury.

[26] Lee CY (2020). Photoacoustic imaging to localize indeterminate pulmonary nodules: a preclinical study. PLoS One.

[27] Stoffels I (2015). Metastatic status of sentinel lymph nodes in melanoma determined noninvasively with multispectral optoacoustic imaging. Sci Transl Med.

[28] Park B (2021). 3D wide-field multispectral photoacoustic imaging of human melanomas in vivo: a pilot study. J Eur Acad Dermatol Venereol.

[29] Hult J (2021). Comparison of photoacoustic imaging and histopathological examination in determining the dimensions of 52 human melanomas and nevi ex vivo. Biomed Opt Express.

[30] He H (2022). Fast raster-scan optoacoustic mesoscopy enables assessment of human melanoma microvasculature in vivo. Nat Commun.

[31] Viator JA (2020). Photoacoustic detection of circulating melanoma cells in late stage patients. J Innov Opt Health Sci.

[32] Galanzha EI (2019). In vivo liquid biopsy using Cytophone platform for photoacoustic detection of circulating tumor cells in patients with melanoma. Sci Transl Med.

[33] Masthoff M (2018). Use of multispectral optoacoustic tomography to diagnose vascular malformations. JAMA Dermatol.

[34] Diot G (2017). Multispectral optoacoustic tomography (MSOT) of human breast cancer. Clin Cancer Res.

[35] Reber J (2018). Non-invasive measurement of brown fat metabolism based on optoacoustic imaging of hemoglobin gradients. Cell Metab.

[36] Erfanzadeh M, Zhu Q (2019). Photoacoustic imaging with low-cost sources; a review. Photoacoustics.

[37] von Knorring T (2025). Normal and melanoma skin visualized, quantified and compared by in vivo photoacoustic imaging. Photoacoustics.

[38] Kukk AF (2024). Non-invasive 3D imaging of human melanocytic lesions by combined ultrasound and photoacoustic tomography: a pilot study. Sci Rep.

[39] Neuwirth M (2017). Detection of melanoma metastases in regional lymph nodes using multispectral photoacoustic imaging. J Am Coll Surg.

[40] D’Mello SA (2016). Signaling pathways in melanogenesis. Int J Mol Sci.

[41] Neuschmelting V (2016). Lymph node micrometastases and in-transit metastases from melanoma: in vivo detection with multispectral optoacoustic imaging in a mouse model. Radiology.

[42] Yin T (2015). Extravascular red blood cells and hemoglobin promote tumor growth and therapeutic resistance as endogenous danger signals. J Immunol.

[43] Cheng SY (1997). Intracerebral tumor-associated hemorrhage caused by overexpression of the vascular endothelial growth factor isoforms VEGF121 and VEGF165 but not VEGF189. Proc Natl Acad Sci U S A.

[44] Chekan EG (2016). Thickness of cadaveric human lung tissue. Surg Technol Int.

[45] Pinkert MA (2018). Review of quantitative multiscale imaging of breast cancer. J Med Imaging (Bellingham).

[46] Weinhardt V (2019). Imaging cell morphology and physiology using X-rays. Biochem Soc Trans.

[47] Strelnikova N (2017). Live cell X-ray imaging of autophagic vacuoles formation and chromatin dynamics in fission yeast. Sci Rep.

[48] Wang Y (2022). Multiscale imaging informs translational mouse modeling of neurological disease. Neuron.

[49] Wu Y, Shroff H (2022). Multiscale fluorescence imaging of living samples. Histochem Cell Biol.

[50] Kennedy GT (2022). Targeted detection of cancer at the cellular level during biopsy by near-infrared confocal laser endomicroscopy. Nat Commun.

[51] Rastinehad AR (2019). Gold nanoshell-localized photothermal ablation of prostate tumors in a clinical pilot device study. Proc Natl Acad Sci U S A.

[52] Chen YS (2019). Miniature gold nanorods for photoacoustic molecular imaging in the second near-infrared optical window. Nat Nanotechnol.

[53] Stritzker J (2013). Vaccinia virus-mediated melanin production allows MR and optoacoustic deep tissue imaging and laser-induced thermotherapy of cancer. Proc Natl Acad Sci U S A.

[54] Zhu J (2022). Photothermal nano-vaccine promoting antigen presentation and dendritic cells infiltration for enhanced immunotherapy of melanoma via transdermal microneedles delivery. Research (wash d c).

[55] Jiang Y (2021). Activatable polymer nanoagonist for second near-infrared photothermal immunotherapy of cancer. Nat Commun.

[56] Chen Q (2016). Photothermal therapy with immune-adjuvant nanoparticles together with checkpoint blockade for effective cancer immunotherapy. Nat Commun.

[57] Esposito M (2024). COL10A1 expression distinguishes a subset of cancer-associated fibroblasts present in the stroma of high-risk basal cell carcinoma. Br J Dermatol.

[58] Gu Z (2016). Complex heatmaps reveal patterns and correlations in multidimensional genomic data. Bioinformatics.

[59] Farshchian M (2015). EphB2 promotes progression of cutaneous squamous cell carcinoma. J Invest Dermatol.

[60] Johnson WE (2007). Adjusting batch effects in microarray expression data using empirical Bayes methods. Biostatistics.

[61] Zhao C (2023). Near-infrared phototheranostic iron pyrite nanocrystals simultaneously induce dual cell death pathways via enhanced Fenton reactions in triple-negative breast cancer. ACS Nano.

[62] (2017). Improving quality of science through better animal welfare: The NC3Rs strategy. Lab Anim.

[63] Davenport ML (2020). Perfusion and inflation of the mouse lung for tumor histology. J Vis Exp.

[64] Haedicke K (2020). High-resolution optoacoustic imaging of tissue responses to vascular-targeted therapies. Nat Biomed Eng.

[65] Nayak TR (2017). Tissue factor-specific ultra-bright SERRS nanostars for Raman detection of pulmonary micrometastases. Nanoscale.

