# Supplementary material for: Label-free streamlined photoacoustic image guidance facilitates NIR-II photoablation in models of melanoma lung metastases
Source: J Clin Invest. 2026 May 1;136(9):e196095. doi: 10.1172/JCI196095 (PMC13132385; doi:10.1172/JCI196095)
Supplement: Supplemental data [file jci-136-196095-s160.pdf]

Label-free streamlined photoacoustic image guidance facilitates NIR-II photoablation in models of melanoma lung metastases

Wei Xing[1,2,#], Yujia Zhou[1,3,#], Katja Haedicke[4], Chenyixin Wang[1], Karla Ximena Vazquez-Prada[4], Hong Wu[1], Zhijun Lin[1], Chrysafis Andreou[4,5], Qize Zhang[4], Ke Shang[6], Ruoyang Hu[6], Moritz Kircher[4,†], Xingdong Ye[7,*], Jan Grimm[4,*], Jiang Yang[1,4, 7,*]

Affiliations:

[1]State Key Laboratory of Oncology in South China, Guangdong Provincial Clinical Research Center for Cancer, Sun Yat-sen University Cancer Center, Guangzhou, 510060, China

[2]Department of Anesthesiology, Sun Yat-sen University Cancer Center, Guangzhou 510060, China

[3]Department of Cardiology, Jiangxi Hypertension Research Institute, The First Affiliated Hospital, Jiangxi Medical College, Nanchang University, Nanchang 330006, China

[4]Department of Radiology, Memorial Sloan Kettering Cancer Center, New York, NY 10065, United States

[5]Department of Electrical and Computer Engineering, University of Cyprus, Nicosia, 1678 Nicosia, Cyprus

[6]Department of Oncology, Xinyang Central Hospital, Xinyang 464000, China

[7]Institute of Dermatology, Guangzhou Dermatology Hospital, Guangzhou Medical University, Guangzhou, 510095, China

[#]These authors contributed equally

[†]Deceased

*Corresponding authors:

yangjiang@sysucc.org.cn; grimmj@mskcc.org; yexingdong@qq.com

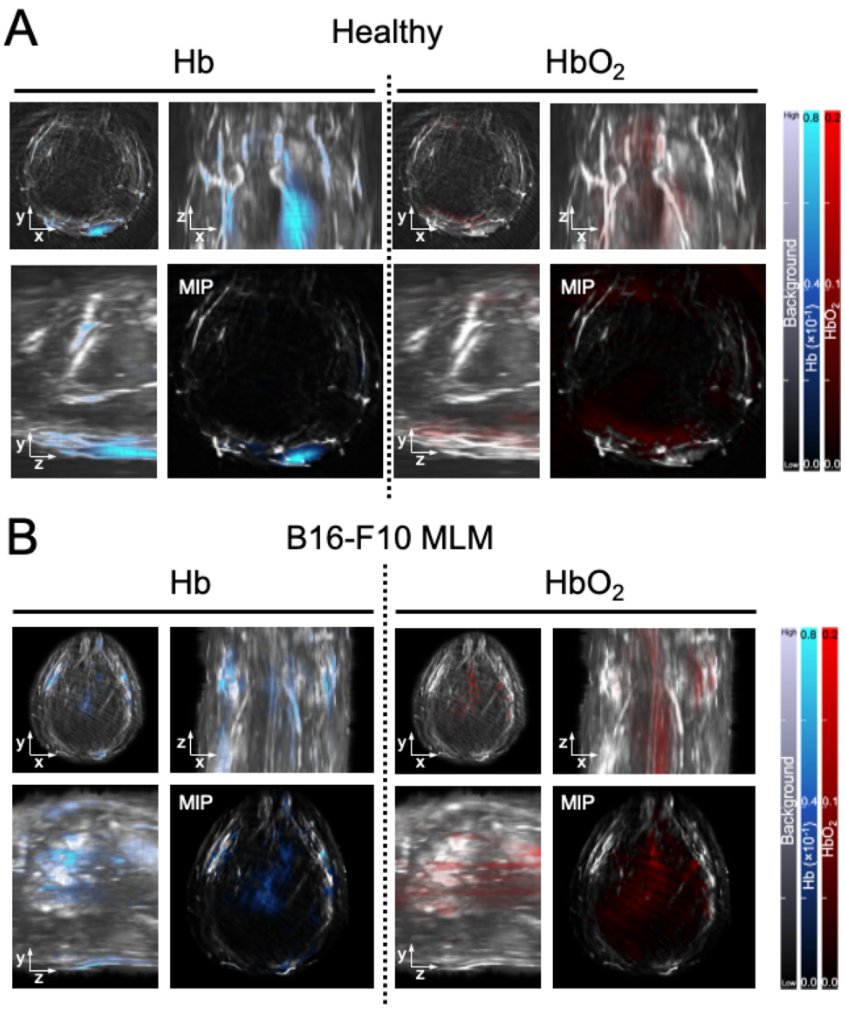

Supplemental Figure 1. Three-dimensional (3D) non-invasive, label-free multispectral optoacoustic tomographic (MSOT) imaging of deoxygenated (Hb) and oxygenated hemoglobin (HbO_2) in (A) healthy and (B) allograft mice bearing B16-F10 melanoma lung metastases (MLM). Specific molecular signals of Hb and HbO_2 are color-coded in blue and red, respectively. Directions of x, y, and z are indicated. Maximum-intensity projection (MIP) images are displayed in the lower-right corner. Associated unmixed mapping images for melanin are shown in Figure 3, D and E.

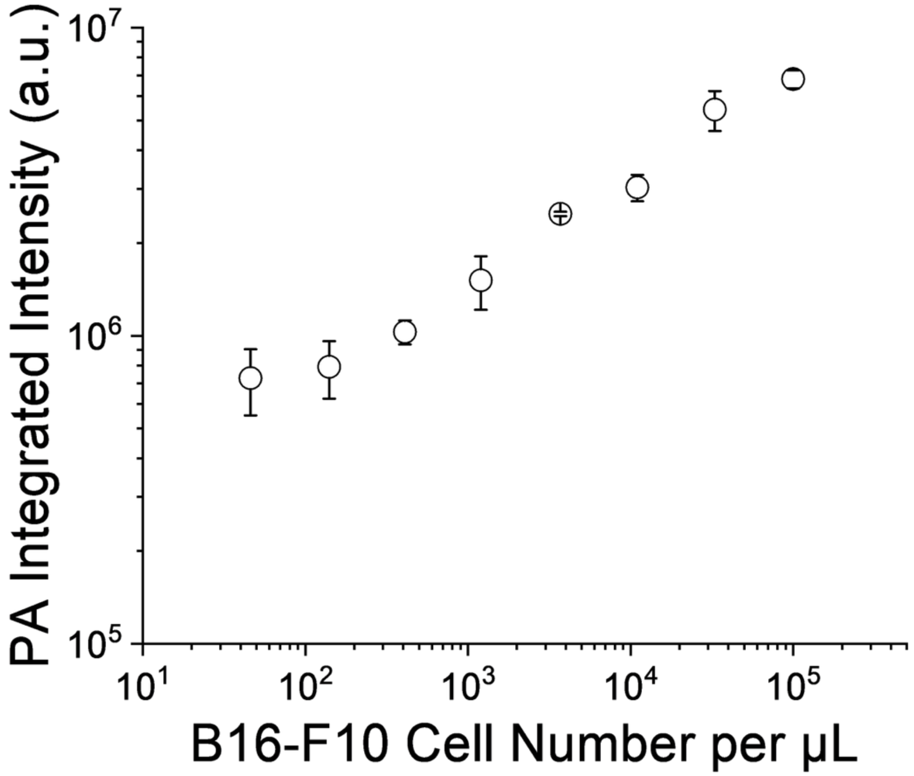

Supplemental Figure 2. Signal quantification for cell imaging phantoms by acoustic-resolution photoacoustic microscopy (AR-PAM) at 808 nm. Phantoms with gradient B16-F10 concentrations are as in Figure 6A.

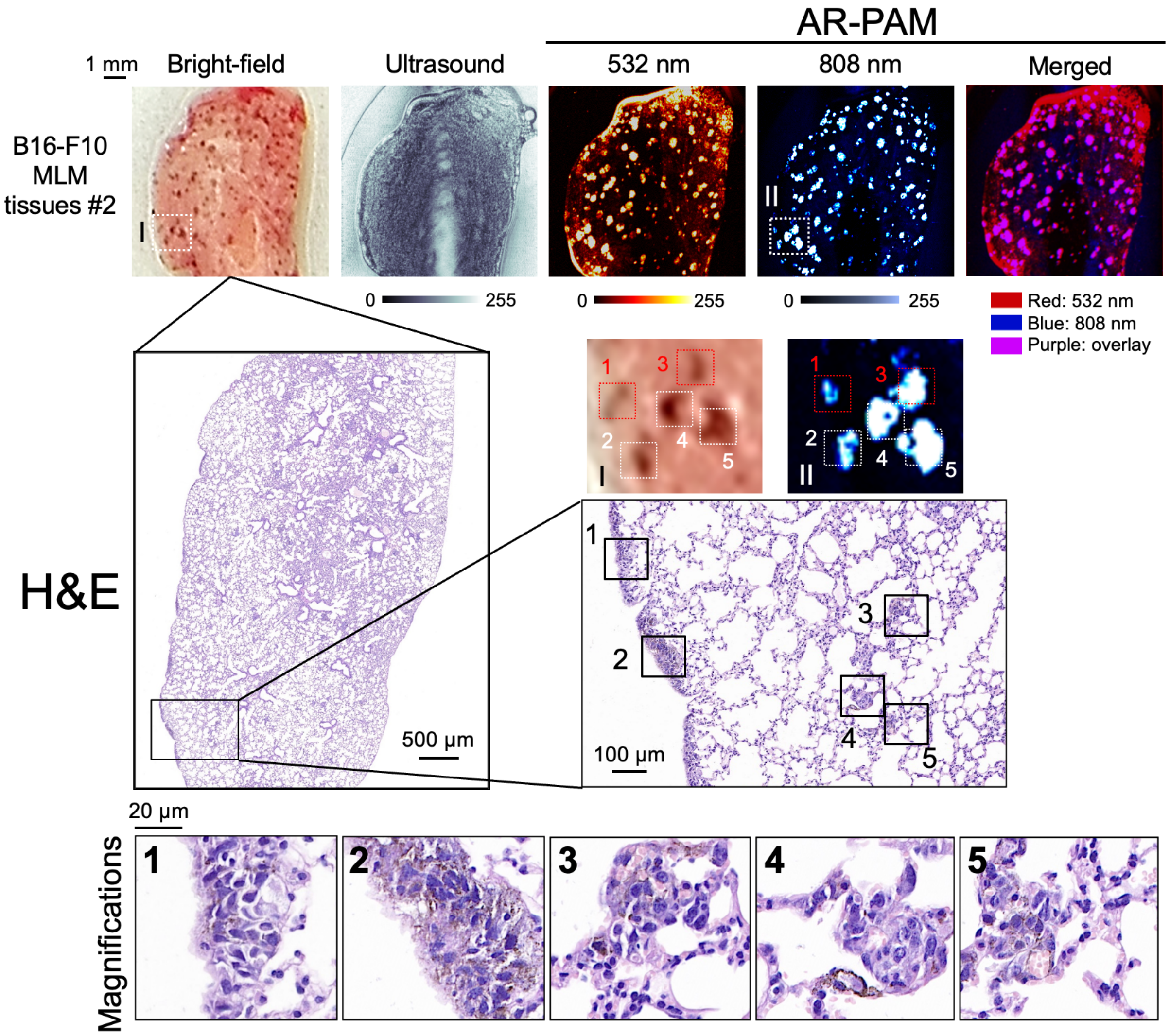

Supplemental Figure 3. AR-PAM images of MLM lung tissues in mouse #2. Ultrasound (US) images are referenced for anatomical registration. Enlarged regions of interest by H&E staining are labeled as indicated and related to photographs and AR-PAM images.

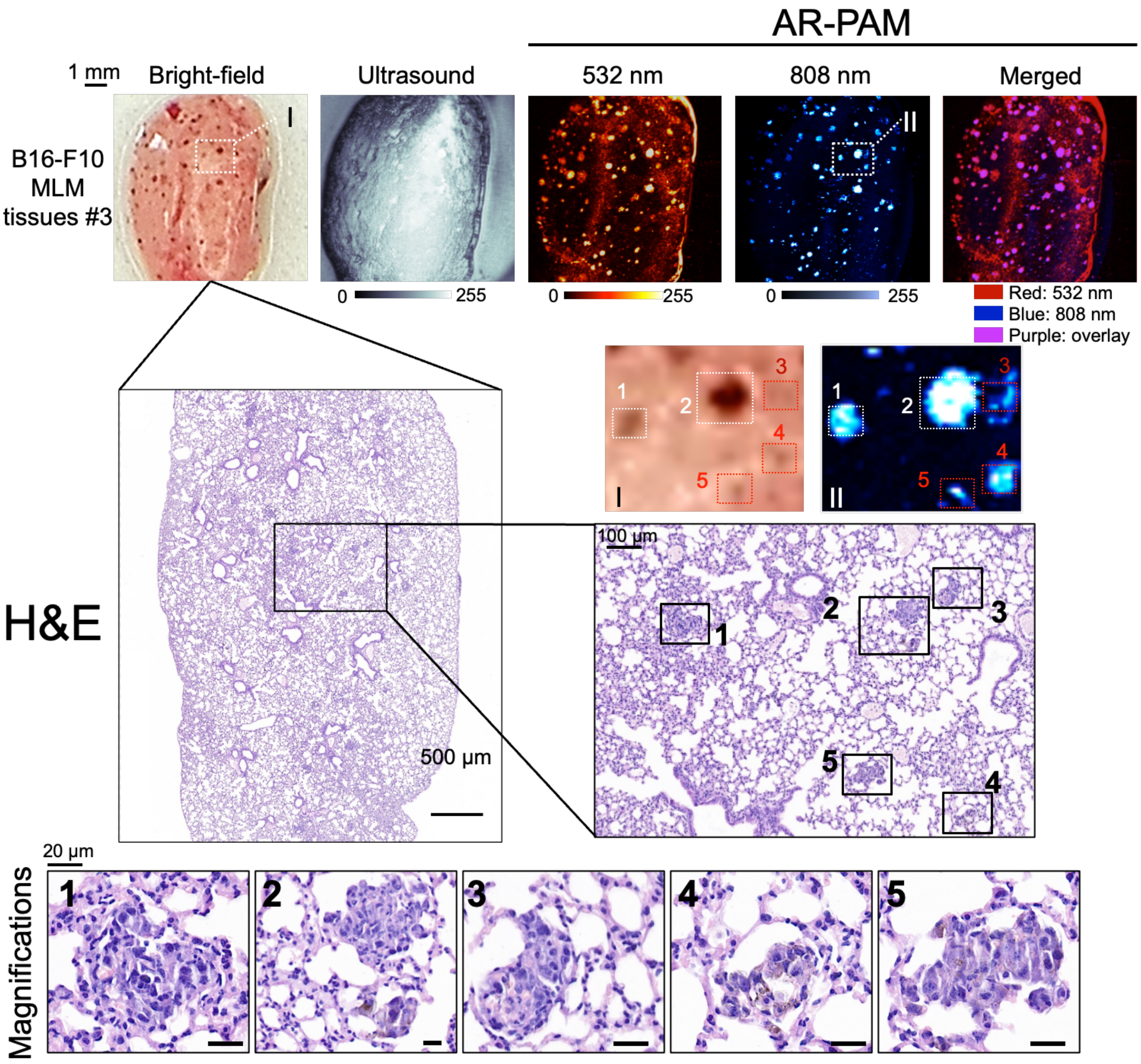

Supplemental Figure 4. AR-PAM images of MLM lung tissues in mouse #3.

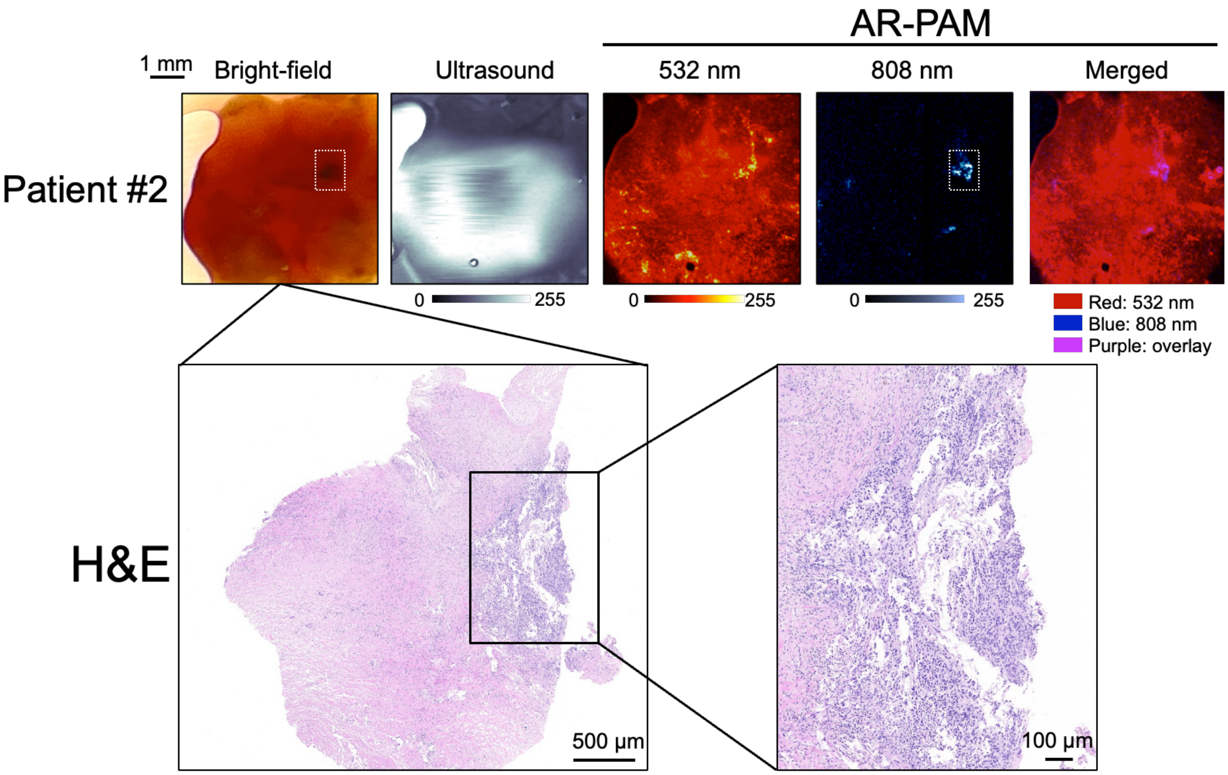

Supplemental Figure 5. AR-PAM images of human MLM lung tissues surgically dissected from patient #2 with a confirmed diagnosis of metastatic melanoma.

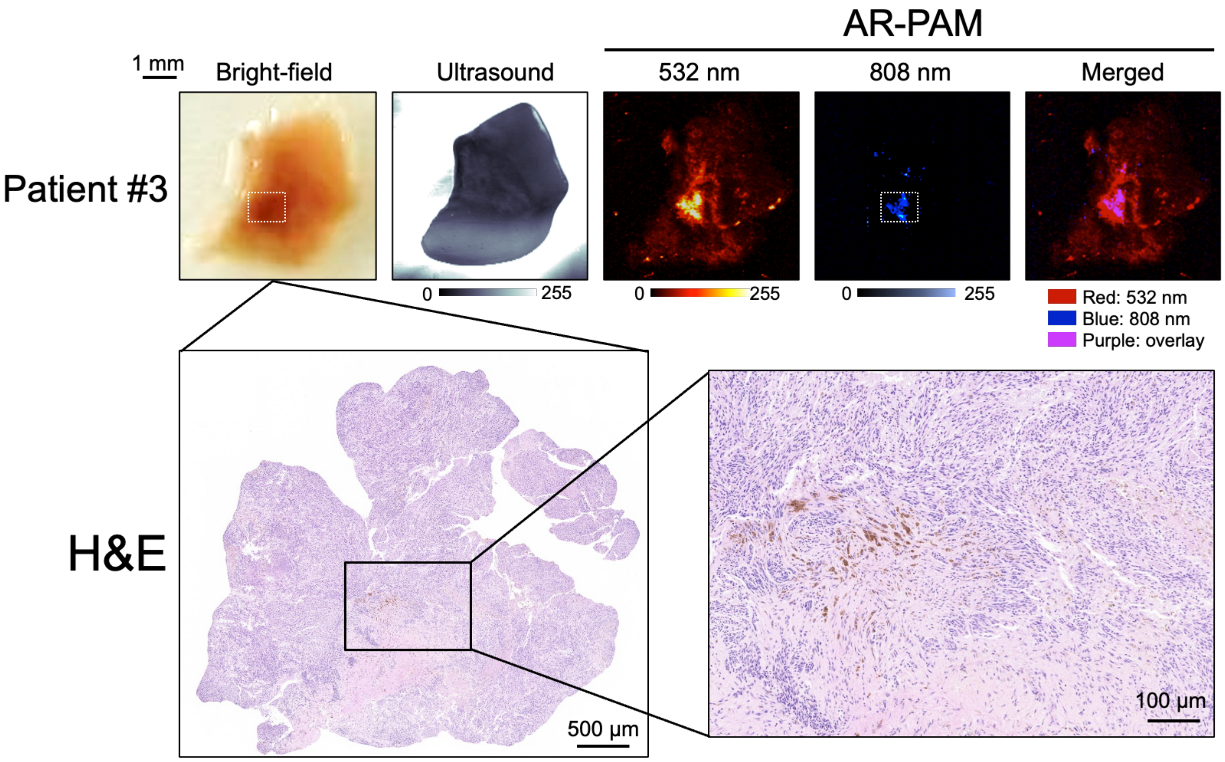

Supplemental Figure 6. AR-PAM images of human MLM lung tissues surgically dissected from patient #3 with a confirmed diagnosis of metastatic melanoma.

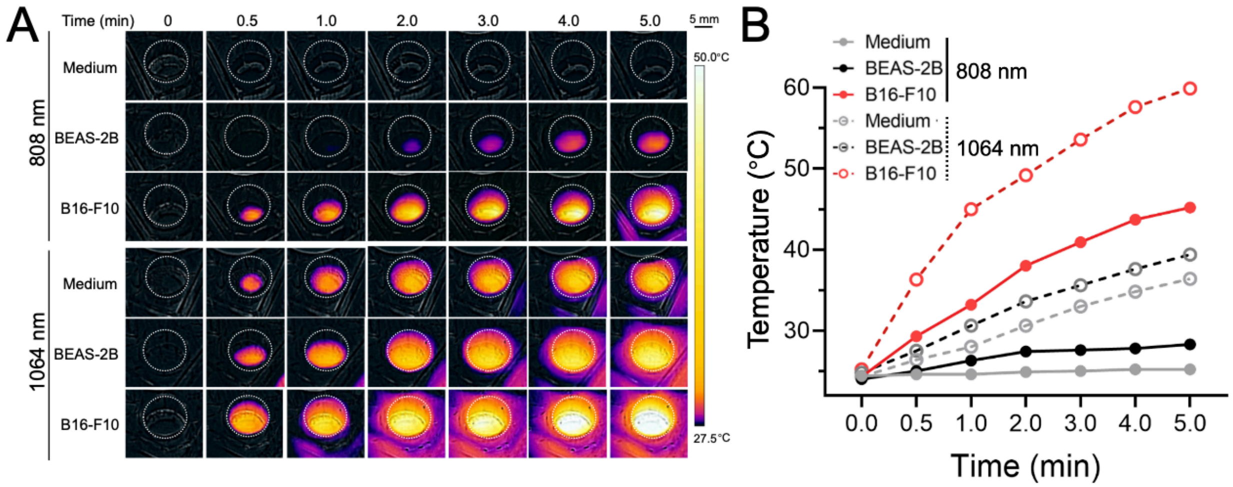

Supplemental Figure 7. In vitro dynamic comparison of NIR-I and NIR-II photoablation. (A) Time-course thermographic phantom imaging and (B) temperature-time dependence curves of blank cell culture medium control, BEAS-2B normal lung epithelial cells, and B16-F10 melanoma cells under 2 W cm^{-2} irradiation power density at 808 nm NIR-I or 1064 nm NIR-II wavelengths.

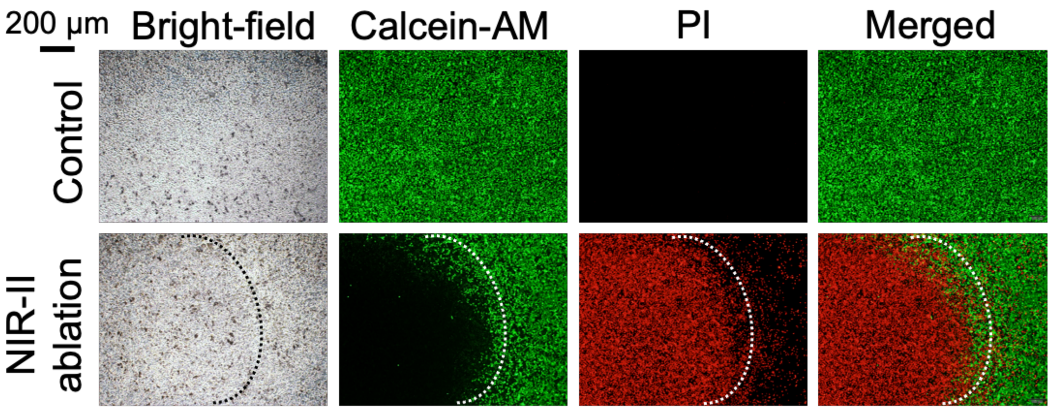

Supplemental Figure 8. Live/dead staining by calcein-AM/propidium iodide (PI) for B16-F10 melanoma cells upon NIR-II photoablation. Nonfluorescent calcein-AM is converted into fluorescent calcein (green) through acetoxymethyl ester hydrolysis by intracellular esterases to indicate live cells, while the red-fluorescent nuclear PI counterstain stains permeable dead cells.

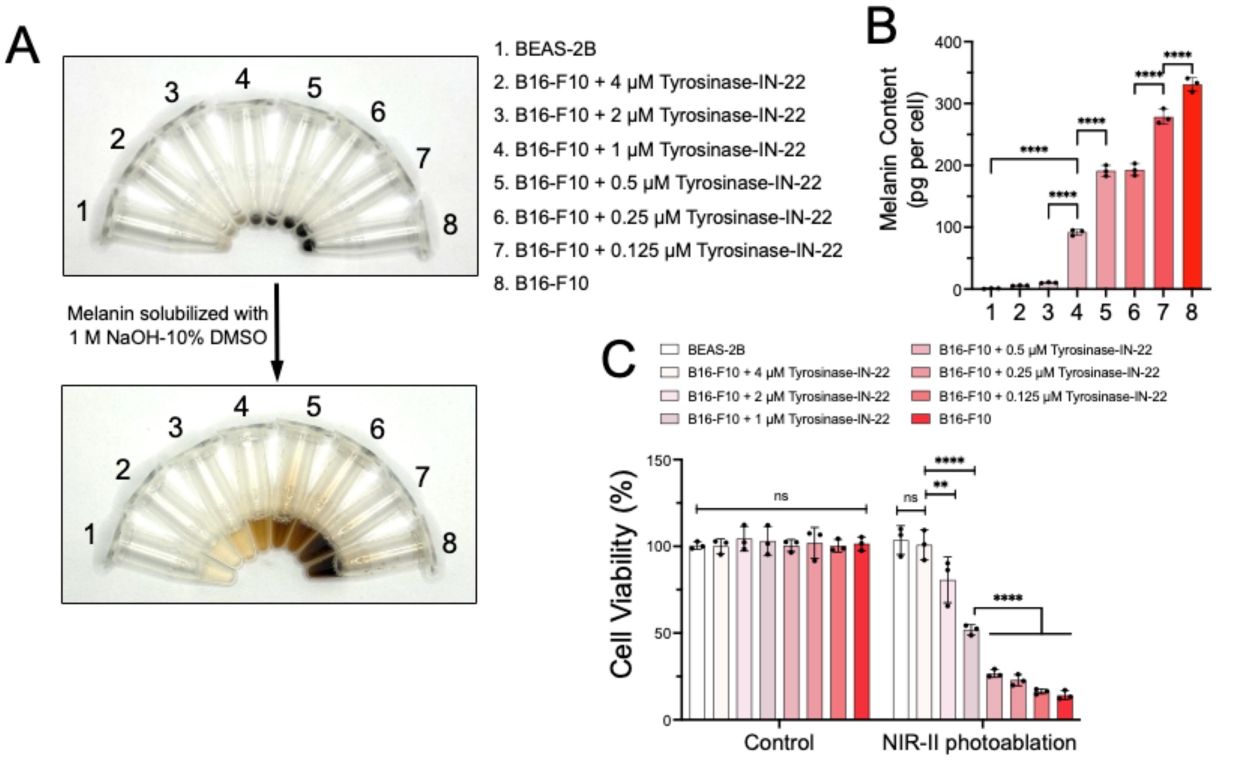

Supplemental Figure 9. Inhibition of melanogenesis in B16-F10 cells by tyrosinase-IN-22. (A) Photographs, (B) melanin content, and (C) cell viability of B16-F10 cells treated with gradient concentrations of tyrosinase-IN-22 (5-chloro-2-mercaptobenzimidazole). BEAS-2B lung epithelial cells were used as a non-melanogenic control. Inhibition of melanogenesis alone did not affect cell viability. A high concentration of 4 μM tyrosinase-IN-22 significantly attenuated the efficacy of photoablation. ** and **** represent statistical significance with *P* values of <0.01 and 0.0001 from Tukey's test, respectively, following a significant one-way ANOVA. Statistical non-significance is denoted as "ns."

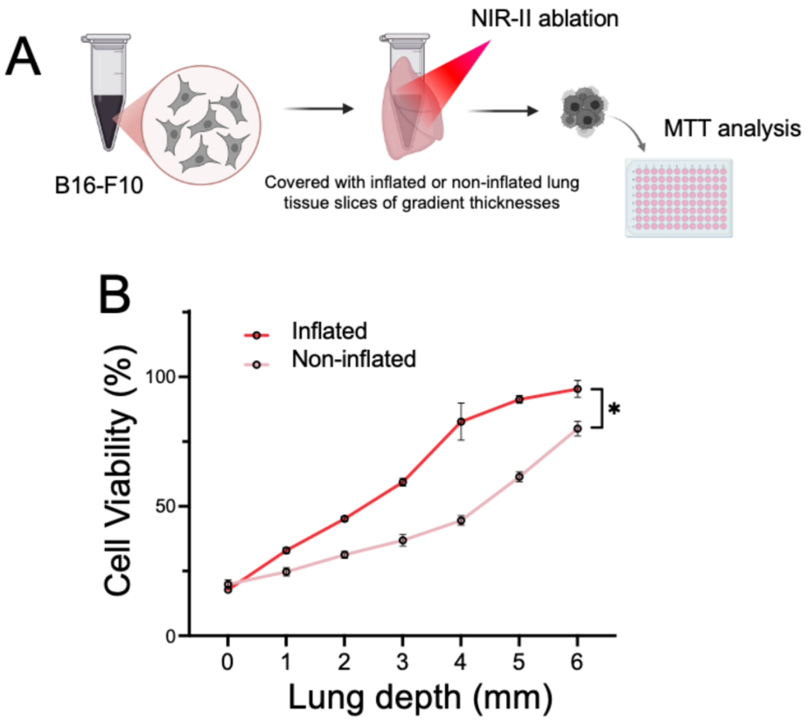

Supplemental Figure 10. NIR-II photothermal ablation of B16-F10 cells in non-inflated and inflated lungs at various tissue depths. (A) Schematic diagram showing the experimental setup to test NIR-II ablation efficacy under inflated or non-inflated states of lungs. (B) Cell viability of B16-F10 cells covered with mouse lung tissues of various thicknesses following 1.5 W cm^{-2} photoablation at 1064 nm for 5 min. * represent statistical significance with a P value of <0.05 from the t-test.

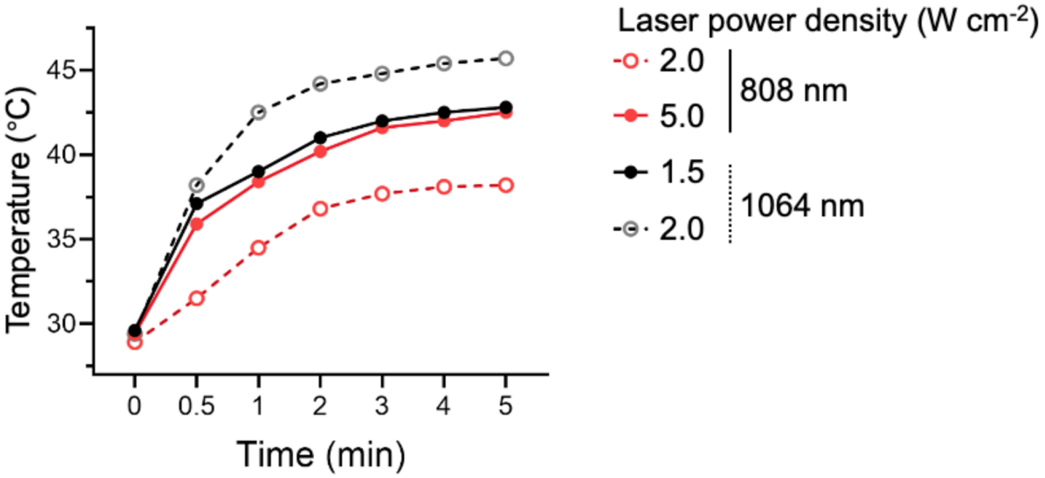

Supplemental Figure 11. Time-course thermographic curves of the lungs in MLM allograft mice receiving NIR-I or NIR-II photoablation at the indicated power densities. Data were quantified from thermographic imaging, as in Fig. 7D.

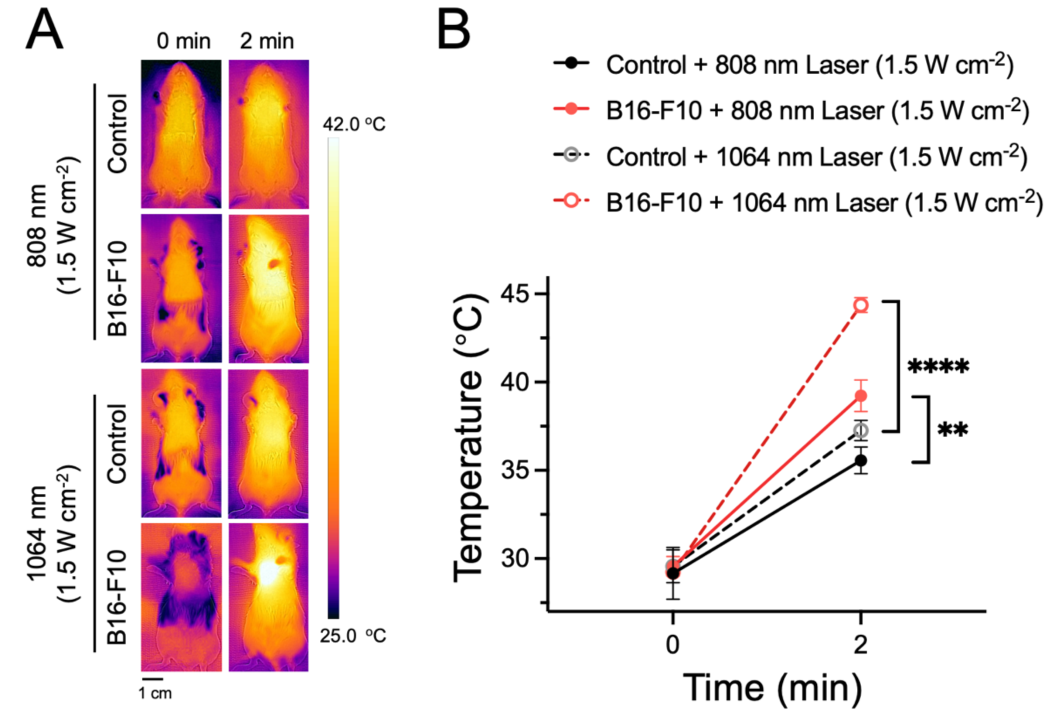

Supplemental Figure 12. Photothermal effects on the lungs of healthy and MLM mice in the near-infrared first (NIR-I, 808 nm) and second (NIR-II, 1064 nm) windows. (A) Thermographic images of healthy and B16-F10 MLM mouse models before and after applications of NIR-I or NIR-II ablation at 1.5 W cm^{-2} for 2 min. (B) Corresponding end-point temperature quantification at the indicated time point following photothermal applications. ** and **** represent statistical significance with *P* values of <0.01 and 0.0001 from the *t*-test, respectively,

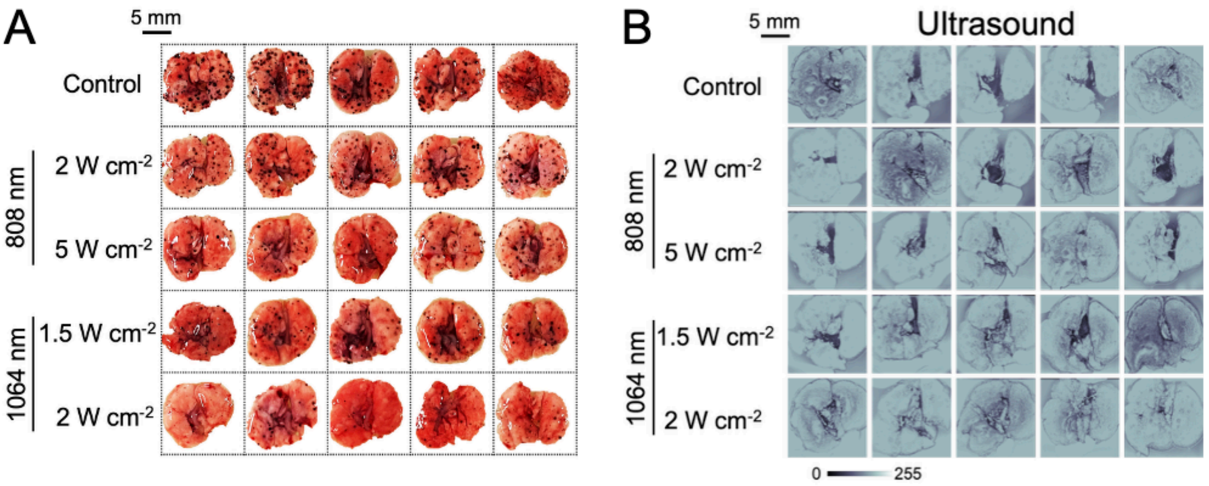

Supplemental Figure 13. Lungs harvested from B16-F10 MLM allograft mice on day 14 after the indicated NIR-I or NIR-II photoablation. (A) Photographs and (B) US images as anatomical references for Figure 7F.

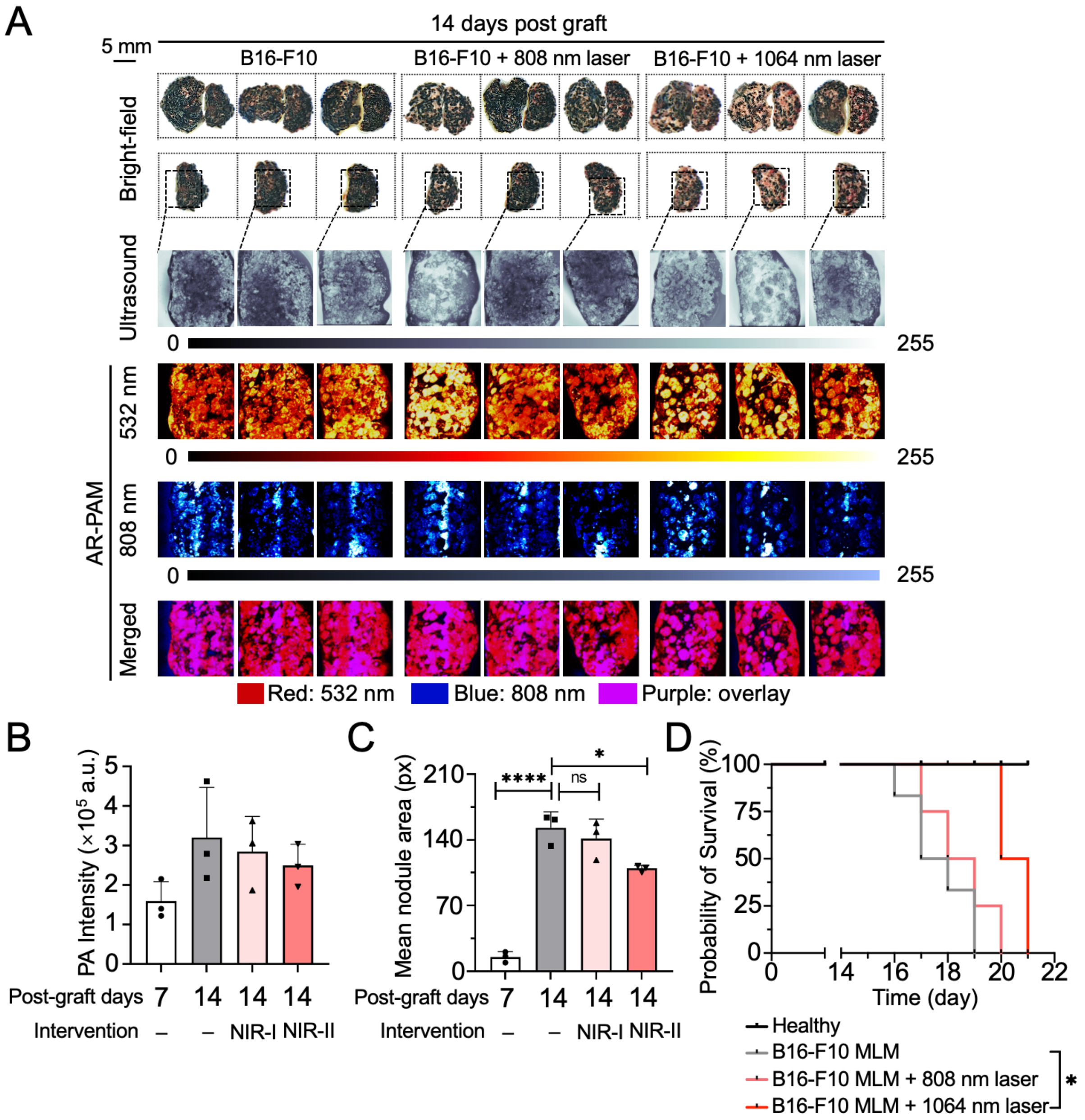

Supplemental Figure 14. Therapeutic monitoring of NIR-II photothermal ablation against advanced-stage MLM by AR-PAM imaging. Twice the melanoma cancer load (2×10^5 cells) was allografted into mice to allow 14 days of tumor progression (twice the growth time). Next, mice received NIR-I or NIR-II ablation at 1.5 W cm^{-2} for 5 min on day 10 post-grafting. (A) Photographs, US, and AR-PAM images of advanced-stage MLM lung tissues on day 14. (B) Image-based PA intensity quantification from AR-PAM images in A. (C) Mean nodule area calculated from bright-field photographs in A. Less advanced MLM lung tissues (1×10^5 cells for 7 days, n=3) from Figure 6D and Supplemental Figures 3 and 4 were included for comparison to denote disease stages. * and **** represent statistical significance with *P* values of <0.05 and 0.0001 from Tukey's test, respectively, following a significant one-way ANOVA. No statistical significance is indicated as "ns". (D) Relative survival probability of advanced-stage MLM mice receiving indicated interventions. Survival curves were compared using a two-sided log-rank test with * indicating a *P* value of < 0.05.

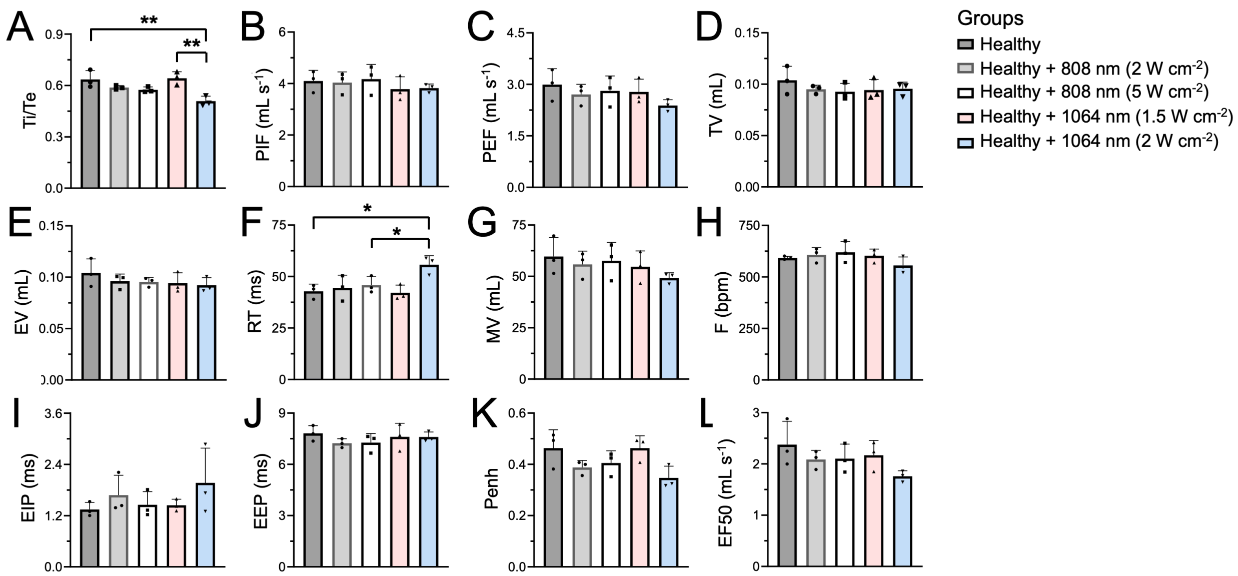

Supplemental Figure 15. Whole-body plethysmography (WBP) for healthy mice on day 14 after receiving NIR-I or NIR-II photoablation intervention at different irradiation power densities (n=3). WBP indices include: (B) The ratio of inspiratory time/expiratory time (Ti/Te), (C) peak inspiratory flow rate (PIF), (D) peak expiratory flow rate (PEF), (E) tidal volume (TV), (F) expiratory volume (EV), (G) relaxation time (RT), (H) minute ventilation volume (MV), (I) respiratory rate (F), (J) end-inspiratory pause (EIP), (K) end-expiratory pause (EEP), (L) bronchoconstriction coefficient (Penh), and (M) forced expiratory flow-50 (EF50) of healthy and MLM allograft mice upon various interventions. * and ** represent statistical significance with *P* values of <0.05 and 0.01 from Tukey's test, respectively, following a significant one-way ANOVA.

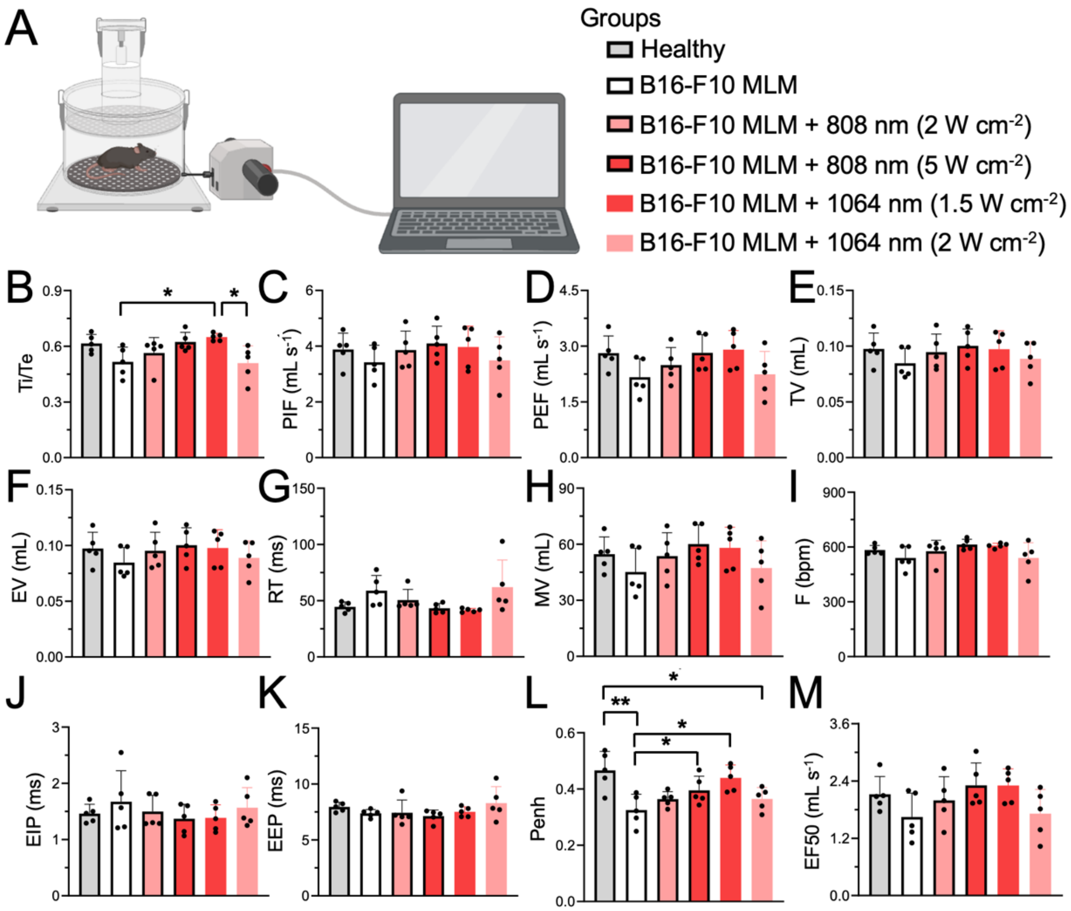

Supplemental Figure 16. WBP for allograft mice bearing B16-F10 MLM on day 14 after receiving NIR-I and NIR-II photoablation at different irradiation power densities. (A) Schematic diagram showing the setup for measuring pulmonary functions using a WBP-4A pulmonary function system. Group assignments are indicated in the right panel for healthy mice and MLM mouse models with allografted B16-F10 tumors that received various ablation regimens (n=5). (B-M) WBP indices are the same as in Supplemental Figure 15. * and ** represent statistical significance with *P* values of <0.05 and 0.01 from Tukey's test, respectively, following a significant one-way ANOVA.

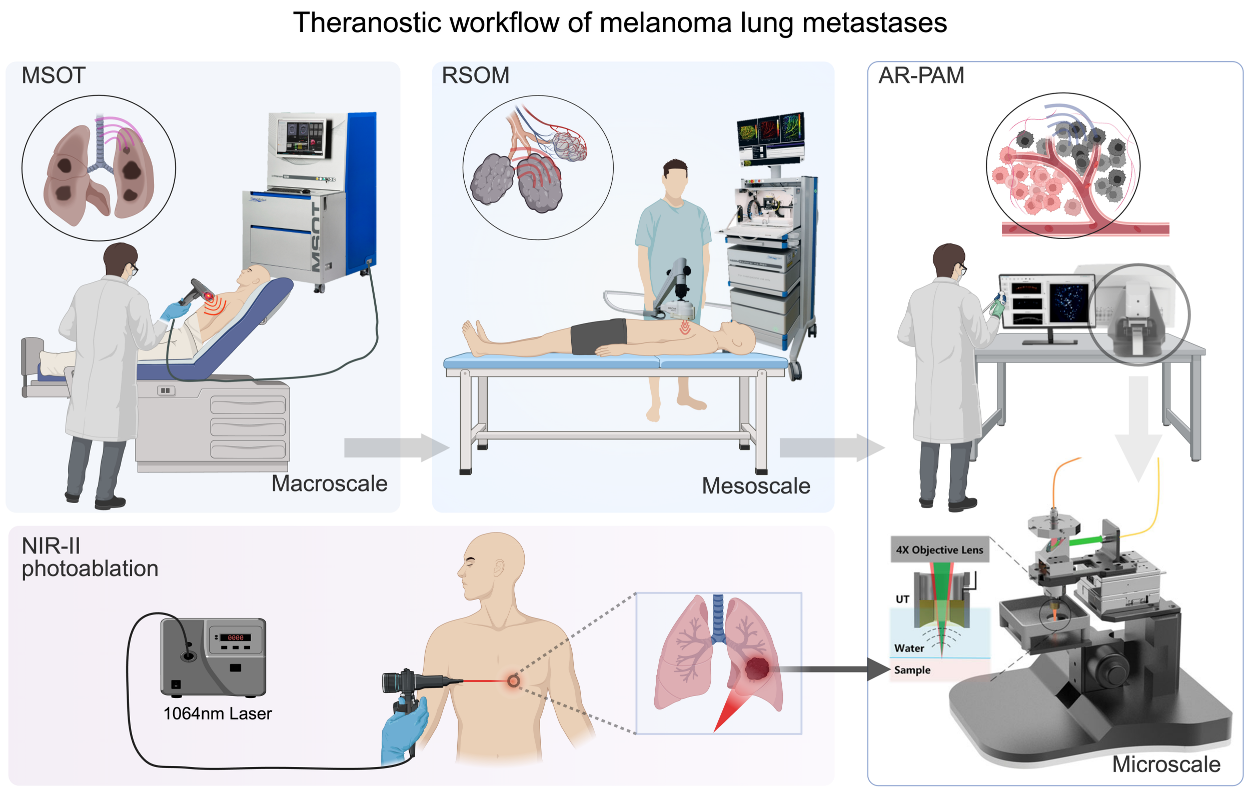

Supplemental Figure 17. Envisaged clinical applications of the theranostic workflow for MLM management during the perioperative period. Rapid preoperative scans can be performed by a radiologist using a handheld MSOT scanner (e.g., the CE-marked MSOT Acuity Echo system). Next, during the minimally invasive procedures, mesoscale scans could be performed to locate tumors. Lastly, integrated bundles of NIR-II ablation and high-resolution AR-PAM could be conducted to eradicate the cancer lesions at histopathological resolution.
